# Scoping review to identify and map the health personnel considered skilled birth attendants in low-and-middle income countries from 2000–2015

**DOI:** 10.1371/journal.pone.0211576

**Published:** 2019-02-01

**Authors:** Amy J. Hobbs, Ann-Beth Moller, Alisa Kachikis, Liliana Carvajal-Aguirre, Lale Say, Doris Chou

**Affiliations:** 1 Department of International Health, The Institute for International Programs, Johns Hopkins Bloomberg School of Public Health, Baltimore, Maryland, United States of America; 2 Department of Reproductive Health and Research, World Health Organization, Geneva, Switzerland; 3 Department of Obstetrics and Gynecology, University of Washington, Seattle, Washington, United States of America; 4 United Nations Children’s Fund, New York City, New York, United States of America; University of Adelaide, AUSTRALIA

## Abstract

**Introduction:**

The “percentage of births attended by a skilled birth attendant" (SBA) is an indicator that has been adopted by several global monitoring frameworks, including the Sustainable Development Goal (SDG) agenda for regular monitoring as part of target 3.1 for reducing maternal mortality by 2030. However, accurate and consistent measurement is challenged by contextual differences between and within countries on the definition of SBA, including the education, training, competencies, and functions they are qualified to perform. This scoping review identifies and maps the health personnel considered SBA in low-to-middle-income-countries (LMIC).

**Methods and analysis:**

A search was conducted inclusive to the years 2000 to 2015 in PubMed/MEDLINE, EMBASE, CINAHL Complete, Cochrane Database of Systematic Reviews, POPLINE and the World Health Organization Global Index Medicus. Original primary source research conducted in LMIC that evaluated the skilled health personnel providing interventions during labour and childbirth were considered for inclusion. All studies reported disaggregated data of SBA cadres and were disaggregated by country.

**Results:**

The search of electronic databases identified a total of 23,743 articles. Overall, 70 articles were included in the narrative synthesis. A total of 102 unique cadres names were identified from 36 LMIC countries. Of the cadres included, 16% represented doctors, 16% were nurses, and 15% were midwives. We found substantial heterogeneity between and within countries on the reported definition of SBA and the education, training, skills and competencies that they were able to perform.

**Conclusion:**

The uncertainty and diversity of reported qualifications and competency of SBA within and between countries requires attention in order to better ascertain strategic priorities for future health system planning, including training and education. These results can inform recommendations around improved coverage measurement and monitoring of SBA moving forward, allowing for more accurate, consistent, and timely data able to guide decisions and action around planning and implementation of maternal and newborn health programmes.

## Introduction

An estimated 303,000 women died from pregnancy or childbirth related complications in 2015 [1] and 2.6 million newborns died in 2016 [2]. Although progress has been made since 1990 [3], maternal and newborn mortality remains a challenge in low- and middle-income countries (LMIC) where there are critical shortages of health personnel who are able to adequately manage and provide quality care during pregnancy and childbirth [1, 4, 5]. The Sustainable Development Goal (SDG) agenda highlights the importance of continued momentum towards improving maternal and newborn health by setting, under the SDG goal 3, targets for achieving a global maternal mortality ratio of less than 70 maternal deaths per 100,000 live births, and aiming for all countries to reduce neonatal mortality to at least as low as 12 per 1,000 live births by 2030 [6, 7].

A key coverage indicator that is included in the SDG Framework is the “proportion of births attended by skilled health personnel” (SDG Indicator 3.1.2) [7, 8]. It has also been identified as a core indicator by other global monitoring frameworks, such as the Global Strategy for Women’s, Adolescent’s, and Children’s Health (GSWACH) (2016–2030) [9], Ending Preventable Maternal Mortality (EPMM) initiative [10] and Every Newborn Action Plan (ENAP) [11]. According to the 2004 joint statement by the World Health Organization (WHO), the International Confederation of Midwives (ICM) and the International Federation of Gynecology and Obstetrics (FIGO), a skilled birth attendant (SBA) is defined as: “*A midwife*, *doctor or nurse—who has been educated and trained to proficiency in the skills needed to manage normal (uncomplicated) pregnancies*, *childbirth and the immediate postnatal period*, *and in the identification*, *management and referral of complications in women and newborns*”[12].

Despite the 2004 WHO/FIGO/ICM joint statement on the definition of a skilled attendant and its core functions, actual reporting at country level from 2000–2015 was challenged by lack of clear guidance on measurement standards and heterogeneity in the use of terminology and cadre functions [13, 14]. Due to this lack of clarity, a 2018 WHO, United Nations Population Fund (UNFPA), United Nations Children’s Fund (UNICEF), ICM, International Council of Nurses (ICN), FIGO and International Paediatric Association (IPA) revision of the definition SBA and further guidance on measurement and monitoring were compiled and updated to align with core maternal and newborn health competencies, education, and regulation of health professionals [15].

The main differences between the 2004 and the 2018 definition of skilled health personnel providing care during labour and childbirth are that, under the revised definition [15], these are health care personnel who can provide effective, uninterrupted and quality care because they are: (a) competent maternal, newborn health (MNH) professionals who hold identified competencies and as a team, these professionals possess all the MNH competencies; (b) are educated, trained and regulated to national and international standards; and (c) are supported within an enabling environment comprising the building blocks of the health system. All competent MNH professionals in a team provide evidence based, human-rights-based, quality, socio-culturally sensitive and dignified care to women, newborns and their families [15]. The scope of the definition of the individual health personnel considered SBA has been broadened outside of the 2004 joint statement definition focusing on cadre names “doctor, nurse, midwife”, in order to encompass other MNH personnel who possess the competencies required to provide intrapartum care, including such providers, in alphabetical order, as anaesthesiologists, doctors (i.e. obstetricians and paediatricians), midwives and nurses [15].

During the Millennium Development Goal (MDG) agenda from 2000–2015 [16], many countries attempted to improve maternal health and survival through task-shifting, increasing the proportion of births attended by skilled health personnel by training lower-level cadre, or creating new cadres able to provide antenatal and intrapartum care [17]. However, the content and requirements of training programs may not be standardized, evaluated, or publicly available. Even when there are practice standards and/or guidelines in place, many countries may lack the capacity and infrastructure needed to adhere to current recommendations for education, training and regulation. Thus, there may be misperception over which health personnel are considered skilled health professionals, what tasks they should be able to perform, how they should be educated and trained, and what health systems infrastructure should be in place in order to support and maintain licensure [18].

The skill level and competencies of cadres may vary, and many cadres that are currently considered 'skilled' may not actually meet the internationally agreed upon definition and criteria set in the 2004 WHO/FIGO/ICM joint statement [13, 18]. These cadre will need continued and expanded monitoring during the SDG timeline to align with the 2018 joint statement [15]. The inclusion of additional country-level health personnel as “skilled” in global monitoring frameworks, without verification of education and training and competencies, coupled with contextual differences between and within countries on the definition of what constitutes a SBA, has complicated the accuracy, comparability, and consistency for continued measurement of SBA moving forward.

The purpose of this scoping review was to identify and map the training, education, skill set, and/or competency of the various cadres of health personnel that provided labour and childbirth care in LMIC during the 2000–2015 MDG era. To our knowledge, no other review has been conducted on this topic previously in the published literature. This information may support the continued refinement and evaluation of the definition of what constitutes a SBA, in order to harmonize and improve the measurement around the global monitoring of SBA coverage and progress of the SDG targets set for 2030 [7].

## Materials and methods

### Study design

The study protocol was published in BMJ Open in 2017 [19]; it outlines an a priori developed and standard methodology for the design and conduct of systematic scoping reviews [20–23]. Arksey and O’Malley’s scoping review framework was applied and the five stages in undertaking this scoping review included: 1) identifying the research question; 2) identifying relevant studies; 3) selecting studies; 4) charting the data; and 5) collating, summarize and reporting the results [20]. This scoping review was conducted to identify, map, summarize, and identify gaps in the existing literature of SBA in LMIC from 2000–2015. Our scoping review was conducted in accordance with the PRISMA (Preferred Reporting Items for Systematic Reviews and Meta-analysis) statement [24] as outlined in S1 Appendix.

### Stage 1: Identifying the research question

All authors included in this review were consulted in identifying and developing the research questions pertaining to this scoping review. The objective of our study was to identify and map cadres considered SBA in relation to education, training, accreditation, certification, legislation, skills, competency; and/or continuing education requirements in LMIC. We sought out to address the following questions:

1. Who are the cadre of health personnel that are reported as ‘skilled birth attendants’ as defined by the 2004 WHO/FIGO/ICM joint statement [12] in LMIC?
2. How do these identified cadres differ between and within county in terms of the following criteria?
  - Curriculum, duration of training and/or education requirements obtained to be qualified;
  - Regulation, accreditation, and/or certification by a professional organization;
  - Skills and competency that each cadre are able to perform (i.e. signal functions and/or other key interventions required for the management of childbirth);
  - Legislation to perform these signal functions and/or interventions;
  - Location of work (urban/rural, hospital/health centre/community-based); and
  - Continuing education requirements (curriculum, duration, and frequency).

### Stage 2: Identifying relevant studies

#### Inclusion criteria

The above captured research questions were assessed and studies were included specific to the following Population, Concept, Study Design and Context criteria presented in Table 1.

**Table 1:** Inclusion criteria for identification of eligible studies.

	Inclusion criteria
**Population**	Any health personnel (paid or voluntary) who provide health services within the provision of maternal and newborn health care during labour and childbirth
**Concept**	Mapping of the health personnel according to education / training received; accreditation or certification; legislation, skills / competency; and/or continuing education requirements
**Study design**	Primary source research of any study design conducted on human subjects (observational studies including prospective or retrospective cohort, case control, and case series; and quasi-experimental; experimental, randomized control trials; and qualitative study designs)
**Context**	Low- and upper-middle-income countries with health facility and/or community-based services offering labour and childbirth care

All primary source study designs reporting on original human subjects research were included if they evaluated the education and/or training received; accreditation or certification requirements; legislation, skills, and/or competencies of the health personnel in order to be considered skilled attendants (paid or voluntary) who provide intrapartum interventions related to the delivery of maternal and newborn health during labour and childbirth. Secondary source data including systematic reviews and other study designs such as case reports, commentaries, editorials, letters, or other opinion pieces were excluded. Research articles reporting on health personnel providing labour and childbirth care were included regardless of whether the cadres were formally regulated as SBA within a country and/or whether they were legislated to perform key health interventions. Studies published inclusive of all languages, any study design, and during 2000 to 2015 in LMIC countries were eligible for inclusion. Countries eligible for inclusion were defined as LMIC according to the World Bank income groupings [25]. Regions were defined and reported according to SDG regional groupings [26].

#### Exclusion criteria

Articles were excluded from the scoping review if:

1. Data were unable to be disaggregated by individual country and/or cadre name, such that there was no mention of the individual health personnel (cadre name) that were considered skilled attendants, providing maternal and newborn care during labour and childbirth, or the country where the cadre were provisioned to provide care; and
2. There were no details in the article of at least one of the following key concepts:
  - Education and/or training requirements that the cadre received to be considered a skilled attendant;
  - Whether the cadre was formally accredited/certified within standard criteria set by the country;
  - Legislation or regulatory requirements; and/or
  - Specific skills or key interventions/signal functions that the cadre was able to perform, regardless of whether they were legislated or authorised.

Personnel who provide supportive care only during labour and childbirth to the woman or act as a birth assistant, such as doulas, were not considered skilled attendants for inclusion in this review. Any study designs evaluating or comparing interventional training programmes were excluded as the intent of our review was to map the training currently available and received by the cadre at the time of the study, not the content of new or existing training programs.

#### Search strategy

The search was conducted for all relevant existing literature without language restrictions based on search terms relating to the research questions restricted to the years 2000–2015, using the following online bibliographic databases: PubMed/MEDLINE, EMBASE, CINAHL Complete, the Cochrane Database of Systematic Reviews, POPLINE, and the WHO Global Index Medicus.

We conducted a manual search of the reference lists of identified studies or systematic reviews as well as a hand search of the grey literature from global initiatives for additional data, including UNFPA, WHO and UNICEF. Reports or published materials beyond those submitted to UNFPA, WHO, or UNICEF were excluded from the search. In order to address which cadres were considered SBA worldwide and what skills they possessed, how they were trained, and how cadres were best supported, a search was conducted with specific terms and Medical Subject Headings (MeSH). An example of the full electronic search strategy performed in PubMed/MEDLINE is outlined in S2 Appendix.

### Stage 3: Study selection

Following the aforementioned comprehensive search strategy, article titles, abstracts, and full-texts were imported into the Covidence platform (https://www.covidence.org), an online-tool developed for systematic reviews in collaboration with the Cochrane Collaboration to screen and assess eligibility of studies for inclusion. Titles and abstracts of each article were independently screened by two reviewers (AJH and ABM; or AJH and LCA). Disagreement between title and abstract screening was resolved by a third reviewer (DC).

Screened abstracts identified for inclusion underwent an independent full-text review by two reviewers (AJH and ABM; AJH and AK; or AJH and LCA), with disagreements resolved by DC. Identified non-English language full-text articles were screened by native/advanced speakers to assess whether they met criteria for inclusion. Full text articles that were excluded at the screening stage had reasons for exclusion documented. The final chosen full-text studies were compared between the reviewers with disagreement being resolved by mutual consensus and with input from all co-authors. Those studies that did not meet the above criteria were excluded and removed from consideration.

### Stage 4 and 5: Charting the data and collating, summarize and reporting the results

Data were extracted using a predetermined template in Microsoft Excel. Information retrieved included study characteristics (country, region, years assessed, study objectives, methodology, and study design). Details about the cadres identified including cadre name, whether they were considered skilled (yes or no), education requirements (entry requirements, duration, or content), continuing education (duration, content, or frequency), reported ability to perform signal functions (perform, sometimes perform, not performed, or not stated) and/or other childbirth interventions (perform, sometimes perform, not performed, or not stated), number of normal or complicated deliveries performed as reported over a defined time period, and whether there were any regulatory bodies or legislation (yes or no) for the cadre named. Classification of cadres as considered “skilled” (yes or no) was based on article report of the cadres as skilled health personnel within the given country; it was not assigned based on author review or interpretation of reported cadre characteristics, education and training, or skills and competencies. In the instance the article did not report any of the above information, including if they were considered skilled, the information was classified as “not stated”.

Data extracted regarding signal functions included the seven basic and the two comprehensive services for emergency obstetric and newborn care (EmONC) as recommended by the WHO [27, 28]. The seven basic signal functions include: 1) administration of intravenous/intramuscular antibiotics; 2) administration of intravenous/intramuscular uterotonic drugs (i.e. oxytocin); 3) administration of intravenous/intramuscular anticonvulsants; 4) manual removal of the placenta; 5) removal of retained products of conception; 6) perform assisted vaginal delivery; and 7) perform basic neonatal resuscitation [27, 28]. The two additional signal functions that comprise comprehensive services include: 8) perform surgery (i.e. caesarean section); and 9) perform blood transfusion [27, 28].

The data extraction form remained flexible for the emergence of other themes and/or categories, which were discussed and developed via consensus from all co-authors, and included additional fields related to SBA, such as evaluation of counseling skills and ability to perform newborn care. Scoping reviews aim to identify and map findings and to provide an overview of the available literature in the topic area rather than an assessment of study quality [23]. Studies were included in this scoping review regardless of their quality. For our review, abstracted quantitative and qualitative data were synthesized using narrative description based on the themes identified.

## Results

### Literature search

**Fig 1:**
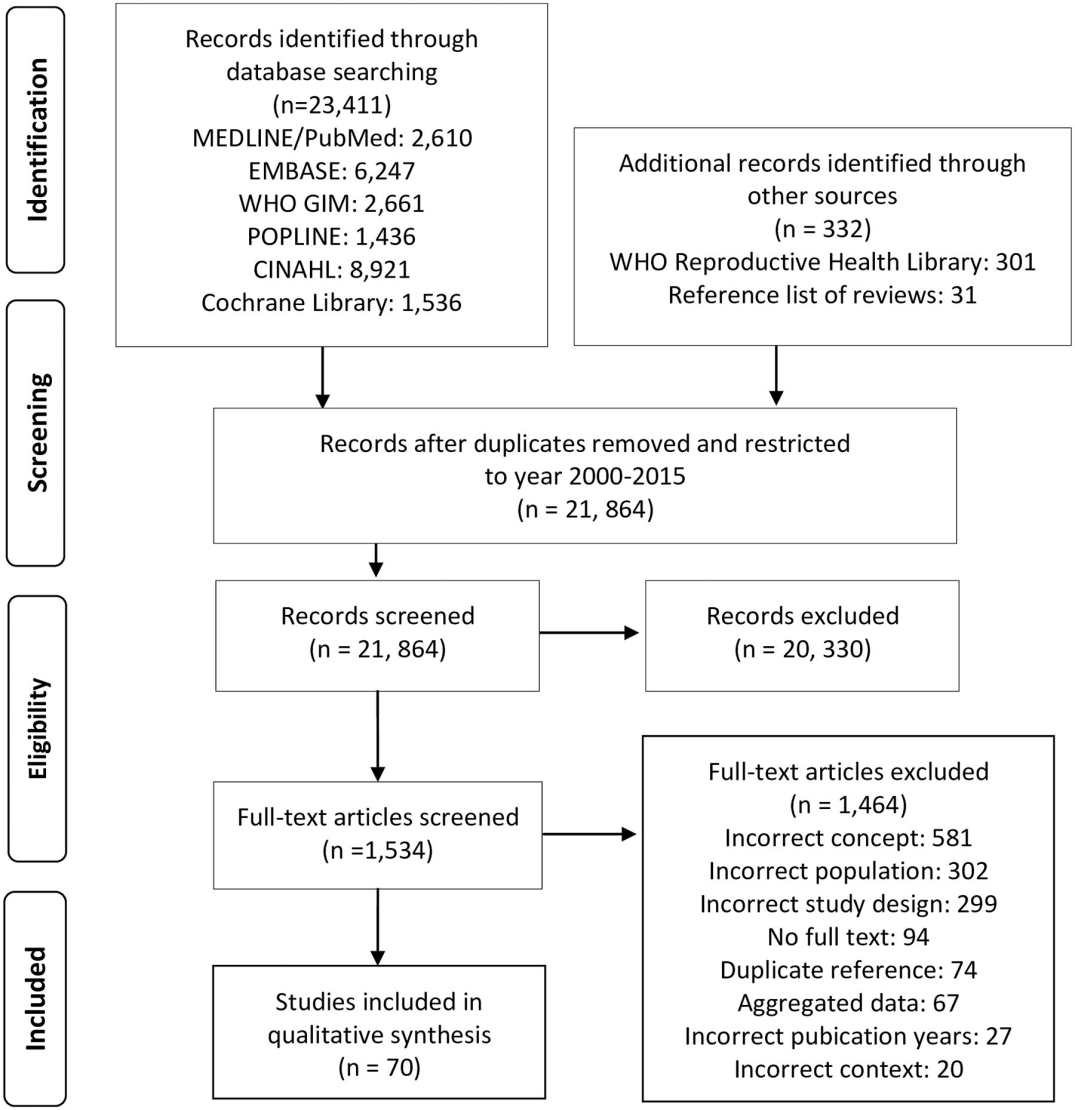
PRISMA literature search and study selection.

The search of electronic databases identified a total of 23,743 articles, comprised of records identified through database searching and hand searching (Fig 1). After removal of duplicates, two reviewers independently screened 21,864 titles and abstracts. A total of 1,534 manuscripts were identified for full text review. After the full text review, 1,464 were excluded. The most common reasons for excluding articles were for “incorrect context”, meaning the study did not provide information about SBA education or training; accreditation or certification; legislation, skills or competency; or continuing education or recertification requirements. The kappa statistic for agreement between authors was unable to be calculated due to the inability of exporting conflicts using the Covidence platform and the large volume of abstracts and full texts screened in this scoping review. Overall, 70 articles were included in our narrative synthesis and are detailed with references in S1 Table. All included studies reported disaggregated data of individual SBA cadres and individual country reported analysis.

### Study characteristics

The study characteristics and cadre information for the 70 articles that were included are shown in Table 2. Included studies were published from 36 different countries. Almost 75% of the included articles were published after the year 2010. Using the SDG region classification [26], over half (57%) of the articles reported on health personnel within sub-Saharan Africa, 27% from Central Asia and Southern Asia and 7% from Latin America and the Caribbean. The majority of countries (69%) in our review had one or two articles included. The countries with three or more studies included investigated approximately 71% of the cadres abstracted. The study designs varied, 57% with cross sectional design, 24% with mixed methods, and 16% with qualitative designs. Only 44% studies reported on a sample of nationally represented data related to SBA cadres. Approximately 40% of included studies presented data from one or two geographic regions, districts, or provinces; or similarly, only one or two hospitals or health facilities within a given country context. Few studies (<20%) reported on SBA cadres working in the community and, only 16% investigated cadres based in predominately rural settings.

**Table 2:** Characteristics of the included studies.

Study Characteristics	Included studies (n = 70)	
	Count (n)	Percent (%)
**Year of Publication**		
2000–2004	4	5.7
2005–2009	15	21.4
2010–2015	51	72.9
**Study design**		
Cohort	1	1.4
Cross-sectional	40	57.1
Mixed methods	17	24.3
Qualitative	11	15.7
Quasi-experimental	1	1.4
**Study setting**		
Rural	11	15.7
Urban	17	24.3
Rural and Urban	33	47.1
Not stated	9	12.9
**Study location**		
Hospital	16	22.9
Health Facility^1^	40	57.1
Community	4	5.7
More than one study location	10	14.3
**Level of representation**		
National	16	22.9
Sub-national	54	77.2
**SDG region**^2^		
Central Asia and Southern Asia	23	26.4
Eastern Asia and South-eastern Asia	4	4.6
Latin America and the Caribbean	7	8.0
Northern America and Europe	1	1.1
Oceania	1	1.1
Sub-Saharan Africa	49	56.3
Western Asia and Northern Africa	2	2.3

^1^ Note that “health facility” includes the following: health facility, health center, health clinic, and/or health post.

^2^ SDG regions presented in the table exclude high-income countries. Note that some studies included more than one country and therefore the count (n = 87) is greater than the number of included studies (n = 70).

### Cadre characteristics

Of the 70 studies included in the review, there were a total of 102 unique cadre names identified, spanning 36 countries. The main cadre names and characteristics of all included cadres are reported in Table 3. A total of 61% of cadres were reported as skilled as stated in the articles included, with 8% not skilled or no consensus on whether considered SBA. Approximately 30% of cadres were unable to be classified as skilled or not, due to lack of report on if they were considered skilled (yes or no) as stated in the article. Of the nearly 30% of cadres that were coded as “other” (i.e. not doctor, nurse, or midwife), nearly half were not considered skilled providers SBA within the country of investigation. Of the 52% of cadres with available data included in the selected articles, nearly 100% reported performing normal deliveries. Legislation status for performing normal deliveries within their country was only stated for 20% of the cadres; 1.5% of evaluated cadres reporting that they performed deliveries even though they were not legislated and 25% of cadres stated that the legislation status was unknown.

**Table 3:** Characteristics of individual cadres included.

Cadre characteristic	Included cadres (n = 341)	
	Frequency (n)	Percent (%)
**Main cadre name categories**		
Doctor	56	16.4
Nurse	56	16.4
Midwife	52	15.3
Nurse Midwife	23	6.7
Auxiliary or Assistant Nurse/Midwife	15	4.4
Other	139	40.8
**Considered skilled?**		
Yes	207	60.7
No	23	6.7
Semi-skilled	5	1.5
No consensus	4	1.2
Not stated	102	29.9
**Perform normal deliveries?**		
Perform	159	46.7
Some perform	15	4.4
Do not perform	2	0.1
Not stated	165	48.4
**Entry education required?**		
Yes	72	21.1
No	2	0.6
Not stated	267	78.3
**Years of education received**		
0–11 months	5	1.5
1–2 years	62	18.2
3–5 years	40	11.7
6–10 years	19	5.6
10+ years	12	3.5
None	23	6.7
Not stated	180	52.8

Nearly half (48%) of the cadre names conformed to the 2004 joint statement internationally agreed upon naming convention of doctor (16%), nurse (16%) or midwife (15%). The remaining cadres were named differently, including 4% for auxiliary or assistant nurses/midwives and 12% for “other” names for nurses, midwives, or nurse/midwives. Categories for “other” doctor, nurse, midwife, or nurse midwives were categorized according to the 2004 joint statement naming conventions for skilled provider (doctor, nurse, midwife) and based on limited evaluation of reported education, training, skills and/or competencies reported for these cadres in the included studies. A detailed breakdown and list of the naming conventions provided in Table 4. The distribution of the number of studies and cadre names published per country, whether or not the cadres were considered SBA, and if the country had education or training details about the listed cadre is found in Table 5. Of the 36 countries that were investigated amongst the 70 studies included in the review, Nigeria had the highest number of studies (n = 11) reporting on the evaluation of most cadres (n = 46).

**Table 4:** List of broad cadre name frequencies and detail from unique cadres identified in the included studies.

Cadre Name	Frequency (n = 341)	Percent (%)	Detailed Cadre Names	Unique Cadres (n = 102)
Clinical Officer	12	3.5	Clinical Officer	1
Clinical Officer—Assistant	4	1.2	Assistant Clinical Officer	1
Doctor	56	16.4	Doctor, Family Doctor, General Doctor, Generalist Physician Obstetrician / Gynaecologist, Obstetrician / Paediatrician, Paediatrician, Resident Doctor, Specialist Physician	9
Doctor—Other	1	0.3	Ayurvedic Doctor	1
Health Worker	17	5.0	Auxiliary Health Worker, Community Health Extension Worker, Family Health Worker, Health Extension Worker, Junior Community Health Extension Worker, Lady Health Worker, Maternal and Child Health Worker, Multipurpose Health Worker (Female)—MPHW (F)/ANM, Senior Auxiliary Health Worker, Senior Community Health Extension Worker	10
Medical Officer	11	3.2	Medical Officer, Medical Officer—Obstetrics trained, Medical Officer of Health	3
Medical Officer—Assistant	2	0.6	Assistant Medical Officer	1
Midwife	52	15.3	Certified Midwife, Midwife, Midwife (CME), Midwife (HIS), Primary Midwife, Professional Midwife, Registered Midwife	7
Midwife—Assistant or Auxiliary	3	0.9	Assistant Midwife, Auxiliary Midwife	2
Midwife—Other	12	3.5	Community Midwife, Enrolled Midwife, Matron, Secondary Midwife, Village Midwife	5
Nurse	56	16.4	Certified Nurse, General Nurse, Neonatal Nurse, Nurse Nurse (BN), Nurse (Generic BSc in Nursing), Nurse (Proficiency Certificate Level), Obstetric Nurse, Public Health Nurse, Registered Community Health Nurse, Registered General Nurse, Registered Nurse, Senior Staff Nurse, Staff Nurse	14
Nurse—Assistant or Auxiliary	6	1.8	Assistant Nurse, Auxiliary Nurse, Nursing Assistant, Nursing Attendant	4
Nurse—Other	12	3.5	Enrolled Nurse, Graduate Nurse, Maternal and Child Health Nurse (B-level), Maternal and Child Health Nurse (Mid-level), Nurse Technician, Nursing Officer, Senior Nursing Sister	7
Nurse Midwife	23	6.7	Nurse Midwife, Registered Nurse Midwife, State Certified Nurse Midwife	3
Nurse Midwife—Auxiliary	6	1.8	Auxiliary Nurse Midwife	1
Nurse Midwife—Other	17	5.0	Community Health Nurse Midwife, Enrolled Nurse Midwife, Nurse Midwife Technician	3
Other	44	12.9	Antenatal Mother, Assistant Health Officer, Community, Health Officer, Community Skilled Birth Attendant, Family Welfare Assistant, Family Welfare Visitor, Feldsher, Female Health Assistant, Female Health Technician, Health Assistant, Health Attendant, Health Officer, Health Orderly, Health Technician, Lady Health Visitor, Maternal and Child Health Aide, Medical Agent (Basic-level), Medical Assistant, Medical Technician (Mid-level), Public Health Officer, Senior Community Health Officer, Senior Health Technician, Senior Nursing Sister, State Enrolled Community Health Nurse, Sub-Assistant Community Medical Officer, Ward Sister	26
Student Doctor, Nurse, or Midwife	4	1.2	Medical Student, Student Doctor (Intern), Student Medical Officer, Student Nurse	4

**Table 5:** Country frequencies for studies included and frequency of cadre names mentioned.

Country	Studies Included		Cadres Included		Average cadres per Study	Skilled^a^ Cadre		Education Details^b^
	Frequency (n = 87)	Percent (%)	Frequency (n = 341)	Percent (%)		Frequency (n = 341)	Percent (%)	
**Afghanistan**	4	5.1	8	2.5	2	8	100	Yes
**Bangladesh**	4	5.1	19	6.1	5	12	63.2	Yes
**Benin**	1	1.3	2	0.6	2	2	100	No
**Cambodia**	1	1.3	4	1.3	4	4	100	Yes
**China**	1	1.3	1	0.3	1	1	100	Yes
**Dominican Republic**	1	1.3	2	0.6	2	2	100	Yes
**Ecuador**	2	2.5	3	1.0	2	3	100	Yes
**Ethiopia**	5	6.3	13	4.1	3	4	30.8	Yes
**Gambia**	1	1.3	9	2.9	9	9	100	No
**Ghana**	4	5.1	12	3.8	3	11	91.7	Yes
**India**	6	7.6	20	6.4	3	16	80.0	Yes
**Indonesia**	1	1.3	6	1.9	6	6	100	Yes
**Jamaica**	1	1.3	4	1.3	4	3	75.0	
**Jordan**	1	1.3	1	0.3	1	1	100	Yes
**Kenya**	1	1.3	10	3.2	10	9	90.0	No
**Kuwait**	1	1.3	1	0.3	1	0	0	No
**Malawi**	4	5.1	24	7.6	6	11	45.8	Yes
**Mali**	3	3.8	15	4.8	5	13	86.7	Yes
**Mexico**	1	1.3	3	1.0	3	3	100	Yes
**Mongolia**	1	1.3	8	2.5	8	4	50.0	Yes
**Mozambique**	1	1.3	6	1.9	6	0	0	Yes
**Nepal**	5	6.3	23	7.3	5	8	34.8	Yes
**Nigeria**	11	13.9	46	14.6	4	17	37.0	Yes
**Pakistan**	4	5.1	22	7.0	6	13	59.1	Yes
**Papua New Guinea**	1	1.3	1	0.3	1	1	100	Yes
**Paraguay**	1	1.3	1	0.3	1	1	100	Yes
**Russia**	1	1.3	2	0.6	2	2	100	Yes
**Rwanda**	1	1.3	3	1.0	3	2	66.7	No
**Sierra Leone**	2	2.5	9	2.9	5	7	77.8	No
**Somalia**	1	1.3	8	2.5	8	7	87.5	No
**South Africa**	1	1.3	1	0.3	1	0	0	No
**Swaziland**	1	1.3	1	0.3	1	0	0	No
**Uganda**	2	2.5	4	1.3	2	0	0	Yes
**United Republic of Tanzania**	7	8.9	40	12.7	6	19	47.5	Yes
**Zambia**	2	2.5	2	0.6	1	1	50.0	No
**Zimbabwe**	2	2.5	7	2.2	4	7	100	No

^a^ Classification of cadres as “skilled” was based on article report of the cadres as skilled health personnel (yes, no) within the given country.

^b^ Refers to article report of care education and/or training details about the cadres investigated within the given country.

### Education and training

Less than 25% of the included cadres in the scoping review reported that there were education or training requirements in order to be considered SBA, with many of these studies reporting within the context of task shifting (e.g. upgrading of competencies of existing cadres). The countries where studies were conducted that mentioned the education or training requirements for the cadre and individual cadre details are found in Table 5 and S1 Table, respectively. Duration of training for SBA varied greatly between and within countries for similar cadres, with some countries reporting as little as 15 weeks of training for a Maternal and Child Health Worker in Nepal [29] and others reporting 10 or more years. Of the cadres with reported education or training details (n = 72), one third (33%) were midwifery cadres with years of training ranging anywhere from 3 months for a Matron in Mali [30] to 10 years for an obstetrician in Nigeria, Sierra Leone, Gambia, Kenya, Malawi, the United Republic of Tanzania, and Zimbabwe [13]. There was wide variation in the duration of training within cadres of the same name, for example 25% were from nursing cadres with training ranging from 18 months after completion of 10^th^ grade secondary schooling for a nurse in Mozambique [31] to 5 years for a bachelors trained nurse in Nepal [32].

None of the articles provided detailed review of curricula in the context of evaluating cadre education and training required to obtain and/or maintain privileging status as a SBA. Due to lack of data reported across the included studies, we were unable to report on whether or not there are requirements in order to maintain competency including licencing and re-licencing of the cadre, such as the number of deliveries attended, percentage of time working in MNH or continuing education. The data did not distinguish between formal education provided and on the job competencies nor were there details on continuing education requirements for refresher or re-training courses in place.

### Skills and competencies

Of the total 341 non-unique cadre names included in this review, less than 40% of studies reported on the cadre’s ability to perform the obstetric and neonatal signal functions [27, 28] to meet the requirements for basic or comprehensive emergency obstetric care (Table 6). Of the articles that reported this information, the majority of cadres (60%) were categorized as being able to perform or sometimes performing the seven basic obstetric and neonatal signal functions. However, only 18% and 11% of cadres, respectively, reported performing all seven BEmONC or both CEmONC functions. Of the cadres that reported being able to perform, less than 1% reported performing the signal functions without the proper legislation. In Nigeria, Junior Community Health Extension Workers and Community Health Extension works were reported as unskilled MNH personnel who were performing the first 5 of the 7 BEmONC signal functions without being legislated to do so [13]. Similarly, Auxiliary Midwives in Nigeria were reported as performing administration of parenteral antibiotics without legislation and there was no consensus from country representatives if they should be classified as SBA. In the same study by Adegoke et al. (2012), Registered Midwives and Registered Nurse Midwives in Sierra Leone were reported as SBA performing manual removal of retained products without being legislated to perform this function. Of the cadres reported as being able and legislated to perform both caesarean sections and blood transfusions, one third stated they were not trained or confident in their ability to conduct these interventions. For example, in the study by Utz et al (2013) [33], in Bangladesh Family Welfare Visitors, Community Skilled Birth Attendants, and Staff Nurses were all reported as being legislated/authorized to perform caesarean sections but acknowledged that they were doing so without receiving the appropriate training. Of the 45 cadres reporting on use of the labour partograph, only 5% reported ability to correctly use this tool and 7% of cadres felt competent to adequately manage postpartum hemorrhage.

**Table 6:** Cadres reported ability to perform basic and comprehensive emergency obstetric and neonatal care (EmONC) signal functions.

Ability to perform	Basic EmONC							Comprehensive EmONC	
	Parenteral antibiotics	Parenteral uterotonic drugs	Parenteral anticonvulsants	Manually removal of placenta	Remove retained products	Assisted vaginal delivery	Perform neonatal resuscitation	Perform surgery (C-section)	Perform blood transfusion
	n (%)	n (%)	n (%)	n (%)	n (%)	n (%)	n (%)	n (%)	n (%)
Perform, legislated	108 (31.7)	102 (29.9)	92 (27.0)	60 (17.6)	53 (15.5)	45 (13.2)	99 (29.0)	33 (9.7)	87 (25.5)
Perform, not legislated	3 (0.9)	2 (0.6)	2 (0.6)	2 (0.6)	2 (0.6)	2 (0.6)	0 (0.0)	0 (0.0)	0 (0.0)
Perform, legislation not stated	25 (7.3)	19 (5.6)	24 (7.0)	30 (8.8)	21 (6.2)	24 (7.0)	17 (5.0)	5 (1.5)	7 (2.1)
Some perform	10 (2.9)	3 (0.87)	6 (1.7)	17 (5.0)	6 (1.8)	5 (1.5)	26 (7.6)	1 (0.3)	11 (3.2)
Not performed	0 (0.0)	8 (2.4)	14 (4.1)	46 (13.5)	51 (14.5)	65 (19.1)	15 (4.4)	85 (24.9)	24 (7.0)
Not stated	195 (57.2)	207 (60.7)	203 (59.5)	183 (53.7)	208 (61.0)	200 (58.7)	184 (54.0)	217 (63.6)	212 (62.2)

## Discussion

To our knowledge, this is the first published scoping review to systematically synthesize the global health workforce in maternal and newborn care in the context of global SBA measurement and monitoring. The key finding from our review suggests that there are large gaps between current evidence-based standards and levels of provider competence within SBA in a given category, workforce, or grouping. Very few articles reported on national level data, making it difficult to evaluate the SBA providers within countries. Given the substantial heterogeneity of SBA cadres within and between countries, it is challenging to summarize differences in how SBAs are defined in LMIC.

Three studies included in this review, authored by Adegoke et al. in 2012 [34], Adegoke et al. 2013 [35] and Utz et al. 2013 [33], contributed to 35% of the cadres listed and 36% of included countries; which led to a high proportion of studies investigating SDG regions [26] in sub-‘Saharan Africa and Central Asia’ and ‘Southern Asia’. Although scoping reviews are not required to include analysis on quality assessment of individual studies, these three studies are exemplary of higher quality national level evaluations of MNH cadre education, training, and competencies in performing labour and childbirth functions. In addition, it is worth noting that several studies included in this review did not report on years of data collection and study setting (e.g. rural and urban), which implicate comparison across studies.

We found a sharp increase in the number of studies included in our review from 2000–2015. This heightened interest corresponds with the inclusion of SBA as a key outcome indicator in the MDGs after the 1999 International Conference on Population and Development (ICPD) [3, 36] and the release of the 2004 WHO/FIGO/ICM joint statement on the definition of the skilled attendant [12]. In our published scoping review protocol [19], we stated that we would compare the cadres included in the scoping review to the 2004 joint statement to assess whether the cadre is considered skilled [12]. Despite guidance from the 2004 joint statement, the cadres presented in over two thirds of the articles did not conform to the standard definition of the skilled attendant (e.g., doctor, nurse, or midwife) as used in the metadata for monitoring MDG Goal 5 [16]. Further, very few studies outlined whether the health personnel working under alternatively named cadre titles possessed the required education and training to meet skills and competencies required to adequately manage or refer for complications related to pregnancy, labour, and childbirth as outlined therein [12].

We also found heterogeneity in reported naming conventions, cadre definitions, sources of data representation (national or sub-national) and a lack of standardized reported education, training and functions performed, and identification of accreditation or regulatory bodies for SBAs in LMIC. The recommendation based on the 2018 joint statement [15], is to go beyond an assessment according to cadre name and instead focus on the core competencies the MNH providers are able to perform for essential care for women and newborns and to take into account a more holistic view, including the enabling environment, the quality of care delivered by a full team of MNH providers, and health system infrastructure, such as service availability and readiness [18].

In addition to assessing provider’s competency to perform key functions, large-scale national evaluations are needed in the future to assess SBAs clinical knowledge and proficiency of technical skills/competencies and health system readiness and availability in accordance with the recommendations outlined in the 2018 joint statement [15]. This should also include an assessment of their understanding of basic and emergency obstetrics intervention. A limitation mentioned in several studies was that while some cadre could perform key functions, they did not know why or when the function should be performed or how to interpret the results, if required, such as an understanding of the pathophysiology or treatment options available [33, 37, 38].

The basic and comprehensive EmONC signal functions and their related indicators are currently being updated by UNPFA, Averting Maternal Death and Disability (AMDD) and WHO in response to improving issues related inexact and inadequate measurement. There has been a preference to report data related to population sizes rather than the number of births and met need for EmONC, as well as defining not only required EmONC facility numbers, but also facility size, capacity and staffing [39]. In addition to core competencies related to obstetric and neonatal emergency signal functions, future evaluations should consider a broader spectrum of competencies such as interpersonal skills and ability to counsel and educate women and their families on relevant topics such as family planning.

There is a large gap between the current state of evidence-based recommendations for SBA and the level of provider competencies reported in the included articles, making it difficult to compare cadres across countries and over time. Few studies mention training and education to be considered SBA, such as the education requirements to enroll in and obtain credentials. Of the studies that did report on educational requirements, the range included big differences in length of study and very few included details on the curriculum. There is a need for standardization of training curriculum and accreditation programs, including licensing and regulatory requirements, continuing education and training, and upgrading or maintaining the competencies of existing SBA cadre. Continuing education or refresher training, consisting of clinical simulation, drills, re-training, supervision and support, are particularly important. Ongoing assessment of requirements to obtain and maintain skills and competencies should be done in order to ensure that the degradation of key functions does not occur over time. Further studies verifying and evaluating country-level capacity to provide adequate continuing education or refresher training is needed in order to provide recommendations to countries with shortages of trained personnel.

For many countries worldwide, the main source of data for the SBA indicator has been from nationally representative population-based household surveys such as the Demographic Health Surveys (DHS) [40] and Multiple Indicator Cluster Surveys (MICS) [41]. Other sources of SBA include routine administrative data from health management information systems reported by Ministries of Health (MoH) and/or National Statistical Offices (NSO) for countries with high level of health information data completeness and high level of health facility utilization. Data shows that countries may not have been consistent in the operationalization of the definition of a 'skilled attendant at birth' as proposed in 2004 WHO/ICM/FIGO joint statement [42]. As a result, the inclusion of additional health personnel as skilled and differences in measurement of SBA over time have affected the comparability, and consistency of measurement of this indicator. In some specific countries, it is difficult to compare the trends of the proportions of each cadre over time with the DHS and MICS data, as the categorization of qualified versus unqualified cadres or inclusion or exclusion of different cadres within the reported categories is not consistent between the surveys conducted. At a country level, the various cadres of health-care providers considered to be “skilled” can change based on new national health policy and/or training programmes as well as task shifting between cadres. The existing data are therefore difficult to interpret and compare (16).

The challenges described above make it difficult to determine the impact towards improving maternal health services, particularly at the time of delivery. In order to improve the measurement coverage of SBA moving forward, it will be important to have a more rigorous customization process at the country level to obtain consensus on reporting skilled health professionals. This would result in increased consistency in measurement and monitoring over time. Improved measurement of SBA should also take into account data triangulation of health facility, routine administrative data, and household surveys to measure beyond provider contact and towards the content and quality of care received [43, 44].

This review is part of the ongoing work for the improvement and standardization of global metrics of the definition and evaluation of SBA for improved comparison within and across countries and regions. Development of accountability with respect to measurement of SBA according to standard education, training, skills and competencies within a given country context, including regulatory frameworks for scope of practice requirements of SBA, is also important for considerations of improved coverage measurement of SBA to ensure better health outcomes for women and newborns.

## Conclusion

This scoping review provides a broad and comprehensive review of currently available literature that verifies country-level definitions and functions of SBA in LMIC. It is clear that there are extensive gaps in the literature that warrant further studies, particularly related to national- and sub-national-level evaluation of SBA related to provider qualifications and competencies. In order to reach the goals and targets of the SDGs, as well as the GSWCAH, EPMM, and ENAP initiatives, women and newborns require competent health professionals to attend to their care during labour and childbirth as well as an enabling environment that allows them to perform their work with the highest quality. Further large scale national evaluations of reported SBA coverage data and meta-data will enable recommendations around improved metrics as well as national and global reporting of this indicator, allowing for appropriate guidance on decisions and action. Improved measurement of SBA in the SDG era and beyond will allow for more accurate, consistent, and timely data able to guide decisions, demand and action around planning and implementation of maternal and newborn health programs.

## Supporting information

S1 Appendix PRISMA (Preferred Reporting Items for Systematic review and Meta-Analysis) 2009 checklist: Recommended items to address in a systematic review. (DOC)

S2 Appendix Skilled birth attendants scoping review search strategy. (DOCX)

S1 Table List and characteristics of included studies. (DOCX)
