# Supplementary material for: Scoping review to identify and map the health personnel considered skilled birth attendants in low-and-middle income countries from 2000–2015
Source: PLoS One. 2019 Feb 1;14(2):e0211576. doi: 10.1371/journal.pone.0211576 (PMC6358074; doi:10.1371/journal.pone.0211576)
Supplement: S2 Appendix — (DOCX) [file pone.0211576.s002.docx]

**S2 Appendix: Skilled birth attendants scoping review search strategy in Pubmed/MEDLINE**

| # | Section | Search Terms |
| --- | --- | --- |
| 1 | Cadre Name | “Health Personnel“[MeSH] OR Midwifery[MeSH] OR Obstetric Nursing[MeSH] OR “Professional Practice”[MeSH] OR “Emergency Responders”[MeSH] OR "Health Occupations"[Mesh] OR "Allied Health Occupations"[Mesh] OR "Students, Health Occupations"[Mesh] OR "Schools, Health Occupations"[Mesh] OR "Health Manpower"[Mesh] OR “health auxiliary”[TIAB] OR “health care manpower”[TIAB] OR “hospital personnel”[TIAB] OR “medical personnel”[TIAB] OR “health care personnel”[TIAB] OR “nursing home personnel”[TIAB] OR “paramedical personnel”[TIAB] OR “Health care workers”[TIAB] OR “Health care worker”[TIAB] OR “Medical Staff”[TIAB] OR “medical personnel”[TIAB] OR “General Practitioners”[TIAB] OR “General Practitioner”[TIAB] OR Caregiver*[TIAB] OR Nurses[TIAB] OR Nurse[TIAB] OR Midwive[TIAB] OR midwives[TIAB] OR midwife[TIAB] OR “Nursing Staff”[TIAB] OR “nurse midwife”[TIAB] OR “nurse midwives”[TIAB] OR “nurse-midwife”[TIAB] OR “nurse-midwives”[TIAB] OR auxiliary[TIAB] OR “Medical students”[TIAB] OR “Nursing students”[TIAB] OR (health*[TI] AND worker*[TI]) OR ”Community Health Workers”[TIAB] OR ”Community Health Worker”[TIAB] OR “Dental Staff”[TIAB] OR doctor[TIAB] OR doctors[TIAB] OR obstetrician[TIAB] OR obstetricians[TIAB] OR “skilled health provider”[TIAB] OR “skilled health providers”[TIAB] OR “skilled attendant”[TIAB] OR “skilled attendants”[TIAB] OR “skilled birth attendant”[TIAB] OR “skilled birth attendants”[TIAB] OR “skilled health”[TIAB] OR “skilled assistance”[TIAB] “skilled assistant”[TIAB] OR “skilled assistants”[TIAB] OR “skilled care”[TIAB] OR “skilled manpower”[TIAB] OR “skilled delivery”[TIAB] OR “skilled staff”[TIAB] OR “skilled person”[TIAB] OR “skilled birth personnel”[TIAB] OR “skilled health workers”[TIAB] OR “skilled health care worker”[TIAB] OR “skilled health care workers”[TIAB] OR “assistance at delivery”[TIAB] OR “assistance at birth”[TIAB] OR “attendant”[TIAB] OR “SAB”[TIAB] OR “SBA”[TIAB] or cadre[TIAB]or cadres[TIAB] OR “health care worker”[TIAB] OR “health care person”[TIAB] OR “health person”[TIAB] OR “health care provider”[TIAB] OR “care provider”[TIAB] OR “care providers”[TIAB] OR “health provider”[TIAB] OR “health providers”[TIAB] OR “health aide”[TIAB] OR “health aides”[TIAB] OR “traditional birth attendant”[TIAB] OR “birth attendant”[TIAB] OR “community birth attendant”[TIAB] OR “health assistant”[TIAB] OR “feldsher”[TIAB] OR “clinical officer”[TIAB] OR “clinical officers”[TIAB] OR “medical officer”[TIAB] OR “medical officers”[TIAB] OR “assistant medical officers”[TIAB] OR “Mother and Child Health Aides”[TIAB] OR “Mother and Child Health Aide”[TIAB] OR “MCHA”[TIAB] OR “mid-level provider”[TIAB] OR “mid-level cadre”[TIAB] OR “mid-level cadres”[TIAB] OR “mid-level healthcare workers”[TIAB] OR “mid-level health care workers”[TIAB] OR “mid-level health workers”[TIAB] OR “community-based skilled birth attendants”[TIAB] OR “community-based skilled birth attendant”[TIAB] OR “community based skilled birth attendants”[TIAB] OR “community based skilled birth attendant”[TIAB] OR “health visitor“[TIAB] or (health*[TI] AND worker*[TI]) OR “fieldworker”[TIAB] OR “task shifting”[TIAB] OR “nonphysicians”[TIAB] OR “non-physician clinicians”[TIAB] OR “non-physician”[TIAB] OR “clinician”[TIAB] OR “clinicians”[TIAB] |
| 2 | Childbirth | “parturition”[MeSH] OR “Delivery, Obstetric”[MeSH] OR “Perinatal Care”[MeSH] OR “Pregnancy Outcome”[MeSH] OR “Pregnancy Complications”[MeSH] OR “Labor, Obstetric”[MeSH] OR “Obstetrics”[MeSH] OR “Postpartum Period”[MeSH] OR “Maternal Health Services”[MeSH] OR "Women's Health Services"[Mesh] OR "Reproductive Health Services"[Mesh] OR “Pregnant Women"[Mesh] OR “Pregnancy"[Mesh] OR “Maternal Death"[Mesh] OR “Maternal-Fetal Relations"[Mesh] OR “Maternal Exposure"[Mesh] OR “Maternal Mortality"[Mesh] OR “Maternal Behavior“[Mesh] OR “pregnant”[TIAB] OR “pregnancy”[TIAB] OR “Maternity”[TIAB] OR “perinatal”[TIAB] OR “peri-natal”[TIAB] OR “peri natal”[TIAB] OR “Postnatal”[TIAB] OR “post natal”[TIAB] OR “post-natal”[TIAB] OR “ante natal”[TIAB] OR “antenatal”[TIAB] OR “ante-natal”[TIAB] OR “Postpartum”[TIAB] OR “Post partum”[TIAB] “Post-partum”[TIAB] OR “puerperium”[TIAB] OR “childbirth care”[TIAB] OR “childbirth”[TIAB] OR “birth”[TIAB] OR “intrapartum”[TIAB] OR (labour[TI] AND delivery[TI]) OR (labor[TI] AND delivery[TI]) OR “maternal health”[TIAB] OR “maternal and child health”[TIAB] OR “maternity care”[TIAB] OR “c-section”[TIAB] OR “caesarean”[TIAB] OR “cesarean”[TIAB] OR “caesarean section”[TIAB] OR “cesarean section”[TIAB] OR “obstetric surgery”[TIAB] OR “signal functions”[TIAB] OR “obstetric interventions”[TIAB] OR “emergency obstetric care”[TIAB] OR “emergency obstetric”[TIAB] OR “emergency obstetrics”[TIAB] OR “EmOC”[TIAB] OR “emergency newborn care”[TIAB] OR “essential obstetric care”[TIAB] OR “vaginal delivery”[TIAB] OR “home birth”[TIAB] or “home births”[TIAB] OR “delivery”[TIAB] OR “deliveries”[TIAB] or “normal delivery”[TIAB] |
| 3 | LMIC – Cochrane Filter | (((((((((Developing Countries[Mesh:noexp] OR Africa[Mesh:noexp] OR Africa, Northern[Mesh:noexp] OR Africa South of the Sahara[Mesh:noexp] OR Africa, Central[Mesh:noexp] OR Africa, Eastern[Mesh:noexp] OR Africa, Southern[Mesh:noexp] OR Africa, Western[Mesh:noexp] OR Asia[Mesh:noexp] OR Asia, Central[Mesh:noexp] OR Asia, Southeastern[Mesh:noexp] OR Asia, Western[Mesh:noexp] OR Caribbean Region[Mesh:noexp] OR West Indies[Mesh:noexp] OR South America[Mesh:noexp] OR Latin America[Mesh:noexp] OR Central America[Mesh:noexp] OR Afghanistan[Mesh:noexp] OR Albania[Mesh:noexp] OR Algeria[Mesh:noexp] OR American Samoa[Mesh:noexp] OR Angola[Mesh:noexp] OR "Antigua and Barbuda"[Mesh:noexp] OR Argentina[Mesh:noexp] OR Armenia[Mesh:noexp] OR Azerbaijan[Mesh:noexp] OR Bahrain[Mesh:noexp] OR Bangladesh[Mesh:noexp] OR Barbados[Mesh:noexp] OR Benin[Mesh:noexp] OR Byelarus[Mesh:noexp] OR Belize[Mesh:noexp] OR Bhutan[Mesh:noexp] OR Bolivia[Mesh:noexp] OR Bosnia-Herzegovina[Mesh:noexp] OR Botswana[Mesh:noexp] OR Brazil[Mesh:noexp] OR Bulgaria[Mesh:noexp] OR Burkina Faso[Mesh:noexp] OR Burundi[Mesh:noexp] OR Cambodia[Mesh:noexp] OR Cameroon[Mesh:noexp] OR Cape Verde[Mesh:noexp] OR Central African Republic[Mesh:noexp] OR Chad[Mesh:noexp] OR Chile[Mesh:noexp] OR China[Mesh:noexp] OR Colombia[Mesh:noexp] OR Comoros[Mesh:noexp] OR Congo[Mesh:noexp] OR Costa Rica[Mesh:noexp] OR Cote d'Ivoire[Mesh:noexp] OR Croatia[Mesh:noexp] OR Cuba[Mesh:noexp] OR Cyprus[Mesh:noexp] OR Czechoslovakia[Mesh:noexp] OR Czech Republic[Mesh:noexp] OR Slovakia[Mesh:noexp] OR Djibouti[Mesh:noexp] OR "Democratic Republic of the Congo"[Mesh:noexp] OR Dominica[Mesh:noexp] OR Dominican Republic[Mesh:noexp] OR East Timor[Mesh:noexp] OR Ecuador[Mesh:noexp] OR Egypt[Mesh:noexp] OR El Salvador[Mesh:noexp] OR Eritrea[Mesh:noexp] OR Estonia[Mesh:noexp] OR Ethiopia[Mesh:noexp] OR Fiji[Mesh:noexp] OR Gabon[Mesh:noexp] OR Gambia[Mesh:noexp] OR "Georgia (Republic)"[Mesh:noexp] OR Ghana[Mesh:noexp] OR Greece[Mesh:noexp] OR Grenada[Mesh:noexp] OR Guatemala[Mesh:noexp] OR Guinea[Mesh:noexp] OR Guinea-Bissau[Mesh:noexp] OR Guam[Mesh:noexp] OR Guyana[Mesh:noexp] OR Haiti[Mesh:noexp] OR Honduras[Mesh:noexp] OR Hungary[Mesh:noexp] OR India[Mesh:noexp] OR Indonesia[Mesh:noexp] OR Iran[Mesh:noexp] OR Iraq[Mesh:noexp] OR Jamaica[Mesh:noexp] OR Jordan[Mesh:noexp] OR Kazakhstan[Mesh:noexp] OR Kenya[Mesh:noexp] OR Korea[Mesh:noexp] OR Kosovo[Mesh:noexp] OR Kyrgyzstan[Mesh:noexp] OR Laos[Mesh:noexp] OR Latvia[Mesh:noexp] OR Lebanon[Mesh:noexp] OR Lesotho[Mesh:noexp] OR Liberia[Mesh:noexp] OR Libya[Mesh:noexp] OR Lithuania[Mesh:noexp] OR Macedonia[Mesh:noexp] OR Madagascar[Mesh:noexp] OR Malaysia[Mesh:noexp] OR Malawi[Mesh:noexp] OR Mali[Mesh:noexp] OR Malta[Mesh:noexp] OR Mauritania[Mesh:noexp] OR Mauritius[Mesh:noexp] OR Mexico[Mesh:noexp] OR Micronesia[Mesh:noexp] OR Middle East[Mesh:noexp] OR Moldova[Mesh:noexp] OR Mongolia[Mesh:noexp] OR Montenegro[Mesh:noexp] OR Morocco[Mesh:noexp] OR Mozambique[Mesh:noexp] OR Myanmar[Mesh:noexp] OR Namibia[Mesh:noexp] OR Nepal[Mesh:noexp] OR Netherlands Antilles[Mesh:noexp] OR New Caledonia[Mesh:noexp] OR Nicaragua[Mesh:noexp] OR Niger[Mesh:noexp] OR Nigeria[Mesh:noexp] OR Oman[Mesh:noexp] OR Pakistan[Mesh:noexp] OR Palau[Mesh:noexp] OR Panama[Mesh:noexp] OR Papua New Guinea[Mesh:noexp] OR Paraguay[Mesh:noexp] OR Peru[Mesh:noexp] OR Philippines[Mesh:noexp] OR Poland[Mesh:noexp] OR Portugal[Mesh:noexp] OR Puerto Rico[Mesh:noexp] OR Romania[Mesh:noexp] OR Russia[Mesh:noexp] OR "Russia (Pre-1917)"[Mesh:noexp] OR Rwanda[Mesh:noexp] OR "Saint Kitts and Nevis"[Mesh:noexp] OR Saint Lucia[Mesh:noexp] OR "Saint Vincent and the Grenadines"[Mesh:noexp] OR Samoa[Mesh:noexp] OR Saudi Arabia[Mesh:noexp] OR Senegal[Mesh:noexp] OR Serbia[Mesh:noexp] OR Montenegro[Mesh:noexp] OR Seychelles[Mesh:noexp] OR Sierra Leone[Mesh:noexp] OR Slovenia[Mesh:noexp] OR Sri Lanka[Mesh:noexp] OR Somalia[Mesh:noexp] OR South Africa[Mesh:noexp] OR Sudan[Mesh:noexp] OR Suriname[Mesh:noexp] OR Swaziland[Mesh:noexp] OR Syria[Mesh:noexp] OR Tajikistan[Mesh:noexp] OR Tanzania[Mesh:noexp] OR Thailand[Mesh:noexp] OR Togo[Mesh:noexp] OR Tonga[Mesh:noexp] OR "Trinidad and Tobago"[Mesh:noexp] OR Tunisia[Mesh:noexp] OR Turkey[Mesh:noexp] OR Turkmenistan[Mesh:noexp] OR Uganda[Mesh:noexp] OR Ukraine[Mesh:noexp] OR Uruguay[Mesh:noexp] OR USSR[Mesh:noexp] OR Uzbekistan[Mesh:noexp] OR Vanuatu[Mesh:noexp] OR Venezuela[Mesh:noexp] OR Vietnam[Mesh:noexp] OR Yemen[Mesh:noexp] OR Yugoslavia[Mesh:noexp] OR Zambia[Mesh:noexp] OR Zimbabwe[Mesh:noexp])) OR (Macedonia[ot] OR Madagascar[ot] OR Malagasy Republic[ot] OR Malaysia[ot] OR Malaya[ot] OR Malay[ot] OR Sabah[ot] OR Sarawak[ot] OR Malawi[ot] OR Nyasaland[ot] OR Mali[ot] OR Malta[ot] OR Marshall Islands[ot] OR Mauritania[ot] OR Mauritius[ot] OR Agalega Islands[ot] OR Mexico[ot] OR Micronesia[ot] OR Middle East[ot] OR Moldova[ot] OR Moldovia[ot] OR Moldovian[ot] OR Mongolia[ot] OR Montenegro[ot] OR Morocco[ot] OR Ifni[ot] OR Mozambique[ot] OR Myanmar[ot] OR Myanma[ot] OR Burma[ot] OR Namibia[ot] OR Nepal[ot] OR Netherlands Antilles[ot] OR New Caledonia[ot] OR Nicaragua[ot] OR Niger[ot] OR Nigeria[ot] OR Northern Mariana Islands[ot] OR Oman[ot] OR Muscat[ot] OR Pakistan[ot] OR Palau[ot] OR Palestine[ot] OR Panama[ot] OR Paraguay[ot] OR Peru[ot] OR Philippines[ot] OR Philipines[ot] OR Phillipines[ot] OR Phillippines[ot] OR Poland[ot] OR Portugal[ot] OR Puerto Rico[ot] OR Romania[ot] OR Rumania[ot] OR Roumania[ot] OR Russia[ot] OR Russian[ot] OR Rwanda[ot] OR Ruanda[ot] OR Saint Kitts[ot] OR St Kitts[ot] OR Nevis[ot] OR Saint Lucia[ot] OR St Lucia[ot] OR Saint Vincent[ot] OR St Vincent[ot] OR Grenadines[ot] OR Samoa[ot] OR Samoan Islands[ot] OR Navigator Island[ot] OR Navigator Islands[ot] OR Sao Tome[ot] OR Saudi Arabia[ot] OR Senegal[ot] OR Serbia[ot] OR Montenegro[ot] OR Seychelles[ot] OR Sierra Leone[ot] OR Slovenia[ot] OR Sri Lanka[ot] OR Ceylon[ot] OR Solomon Islands[ot] OR Somalia[ot] OR Sudan[ot] OR Suriname[ot] OR Surinam[ot] OR Swaziland[ot] OR Syria[ot] OR Tajikistan[ot] OR Tadzhikistan[ot] OR Tadjikistan[ot] OR Tadzhik[ot] OR Tanzania[ot] OR Thailand[ot] OR Togo[ot] OR Togolese Republic[ot] OR Tonga[ot] OR Trinidad[ot] OR Tobago[ot] OR Tunisia[ot] OR Turkey[ot] OR Turkmenistan[ot] OR Turkmen[ot] OR Uganda[ot] OR Ukraine[ot] OR Uruguay[ot] OR USSR[ot] OR Soviet Union[ot] OR Union of Soviet Socialist Republics[ot] OR Uzbekistan[ot] OR Uzbek OR Vanuatu[ot] OR New Hebrides[ot] OR Venezuela[ot] OR Vietnam[ot] OR Viet Nam[ot] OR West Bank[ot] OR Yemen[ot] OR Yugoslavia[ot] OR Zambia[ot] OR Zimbabwe[ot] OR Rhodesia[ot])) OR (Africa[ot] OR Asia[ot] OR Caribbean[ot] OR West Indies[ot] OR South America[ot] OR Latin America[ot] OR Central America[ot] OR Afghanistan[ot] OR Albania[ot] OR Algeria[ot] OR Angola[ot] OR Antigua[ot] OR Barbuda[ot] OR Argentina[ot] OR Armenia[ot] OR Armenian[ot] OR Aruba[ot] OR Azerbaijan[ot] OR Bahrain[ot] OR Bangladesh[ot] OR Barbados[ot] OR Benin[ot] OR Byelarus[ot] OR Byelorussian[ot] OR Belarus[ot] OR Belorussian[ot] OR Belorussia[ot] OR Belize[ot] OR Bhutan[ot] OR Bolivia[ot] OR Bosnia[ot] OR Herzegovina[ot] OR Hercegovina[ot] OR Botswana[ot] OR Brasil[ot] OR Brazil[ot] OR Bulgaria[ot] OR Burkina Faso[ot] OR Burkina Fasso[ot] OR Upper Volta[ot] OR Burundi[ot] OR Urundi[ot] OR Cambodia[ot] OR Khmer Republic[ot] OR Kampuchea[ot] OR Cameroon[ot] OR Cameroons[ot] OR Cameron[ot] OR Camerons[ot] OR Cape Verde[ot] OR Central African Republic[ot] OR Chad[ot] OR Chile[ot] OR China[ot] OR Colombia[ot] OR Comoros[ot] OR Comoro Islands[ot] OR Comores[ot] OR Mayotte[ot] OR Congo[ot] OR Zaire[ot] OR Costa Rica[ot] OR Cote d'Ivoire[ot] OR Ivory Coast[ot] OR Croatia[ot] OR Cuba[ot] OR Cyprus[ot] OR Czechoslovakia[ot] OR Czech Republic[ot] OR Slovakia[ot] OR Slovak Republic[ot] OR Djibouti[ot] OR French Somaliland[ot] OR Dominica[ot] OR Dominican Republic[ot] OR East Timor[ot] OR East Timur[ot] OR Timor Leste[ot] OR Ecuador[ot] OR Egypt[ot] OR United Arab Republic[ot] OR El Salvador[ot] OR Eritrea[ot] OR Estonia[ot] OR Ethiopia[ot] OR Fiji[ot] OR Gabon[ot] OR Gabonese Republic[ot] OR Gambia[ot] OR Gaza[ot] OR "Georgia Republic"[ot] OR "Georgian Republic"[ot] OR Ghana[ot] OR Gold Coast[ot] OR Greece[ot] OR Grenada[ot] OR Guatemala[ot] OR Guinea[ot] OR Guam[ot] OR Guiana[ot] OR Guyana[ot] OR Haiti[ot] OR Honduras[ot] OR Hungary[ot] OR India[ot] OR Maldives[ot] OR Indonesia[ot] OR Iran[ot] OR Iraq[ot] OR Isle of Man[ot] OR Jamaica[ot] OR Jordan[ot] OR Kazakhstan[ot] OR Kazakh[ot] OR Kenya[ot] OR Kiribati[ot] OR Korea[ot] OR Kosovo[ot] OR Kyrgyzstan[ot] OR Kirghizia[ot] OR Kyrgyz Republic[ot] OR Kirghiz[ot] OR Kirgizstan[ot] OR "Lao PDR"[ot] OR Laos[ot] OR Latvia[ot] OR Lebanon[ot] OR Lesotho[ot] OR Basutoland[ot] OR Liberia[ot] OR Libya[ot] OR Lithuania[ot])) OR (Macedonia[tiab] OR Madagascar[tiab] OR Malagasy Republic[tiab] OR Malaysia[tiab] OR Malaya[tiab] OR Malay[tiab] OR Sabah[tiab] OR Sarawak[tiab] OR Malawi[tiab] OR Nyasaland[tiab] OR Mali[tiab] OR Malta[tiab] OR Marshall Islands[tiab] OR Mauritania[tiab] OR Mauritius[tiab] OR Agalega Islands[tiab] OR Mexico[tiab] OR Micronesia[tiab] OR Middle East[tiab] OR Moldova[tiab] OR Moldovia[tiab] OR Moldovian[tiab] OR Mongolia[tiab] OR Montenegro[tiab] OR Morocco[tiab] OR Ifni[tiab] OR Mozambique[tiab] OR Myanmar[tiab] OR Myanma[tiab] OR Burma[tiab] OR Namibia[tiab] OR Nepal[tiab] OR Netherlands Antilles[tiab] OR New Caledonia[tiab] OR Nicaragua[tiab] OR Niger[tiab] OR Nigeria[tiab] OR Northern Mariana Islands[tiab] OR Oman[tiab] OR Muscat[tiab] OR Pakistan[tiab] OR Palau[tiab] OR Palestine[tiab] OR Panama[tiab] OR Paraguay[tiab] OR Peru[tiab] OR Philippines[tiab] OR Philipines[tiab] OR Phillipines[tiab] OR Phillippines[tiab] OR Poland[tiab] OR Portugal[tiab] OR Puerto Rico[tiab] OR Romania[tiab] OR Rumania[tiab] OR Roumania[tiab] OR Russia[tiab] OR Russian[tiab] OR Rwanda[tiab] OR Ruanda[tiab] OR Saint Kitts[tiab] OR St Kitts[tiab] OR Nevis[tiab] OR Saint Lucia[tiab] OR St Lucia[tiab] OR Saint Vincent[tiab] OR St Vincent[tiab] OR Grenadines[tiab] OR Samoa[tiab] OR Samoan Islands[tiab] OR Navigator Island[tiab] OR Navigator Islands[tiab] OR Sao Tome[tiab] OR Saudi Arabia[tiab] OR Senegal[tiab] OR Serbia[tiab] OR Montenegro[tiab] OR Seychelles[tiab] OR Sierra Leone[tiab] OR Slovenia[tiab] OR Sri Lanka[tiab] OR Ceylon[tiab] OR Solomon Islands[tiab] OR Somalia[tiab] OR Sudan[tiab] OR Suriname[tiab] OR Surinam[tiab] OR Swaziland[tiab] OR Syria[tiab] OR Tajikistan[tiab] OR Tadzhikistan[tiab] OR Tadjikistan[tiab] OR Tadzhik[tiab] OR Tanzania[tiab] OR Thailand[tiab] OR Togo[tiab] OR Togolese Republic[tiab] OR Tonga[tiab] OR Trinidad[tiab] OR Tobago[tiab] OR Tunisia[tiab] OR Turkey[tiab] OR Turkmenistan[tiab] OR Turkmen[tiab] OR Uganda[tiab] OR Ukraine[tiab] OR Uruguay[tiab] OR USSR[tiab] OR Soviet Union[tiab] OR Union of Soviet Socialist Republics[tiab] OR Uzbekistan[tiab] OR Uzbek OR Vanuatu[tiab] OR New Hebrides[tiab] OR Venezuela[tiab] OR Vietnam[tiab] OR Viet Nam[tiab] OR West Bank[tiab] OR Yemen[tiab] OR Yugoslavia[tiab] OR Zambia[tiab] OR Zimbabwe[tiab] OR Rhodesia[tiab])) OR (Africa[tiab] OR Asia[tiab] OR Caribbean[tiab] OR West Indies[tiab] OR South America[tiab] OR Latin America[tiab] OR Central America[tiab] OR Afghanistan[tiab] OR Albania[tiab] OR Algeria[tiab] OR Angola[tiab] OR Antigua[tiab] OR Barbuda[tiab] OR Argentina[tiab] OR Armenia[tiab] OR Armenian[tiab] OR Aruba[tiab] OR Azerbaijan[tiab] OR Bahrain[tiab] OR Bangladesh[tiab] OR Barbados[tiab] OR Benin[tiab] OR Byelarus[tiab] OR Byelorussian[tiab] OR Belarus[tiab] OR Belorussian[tiab] OR Belorussia[tiab] OR Belize[tiab] OR Bhutan[tiab] OR Bolivia[tiab] OR Bosnia[tiab] OR Herzegovina[tiab] OR Hercegovina[tiab] OR Botswana[tiab] OR Brasil[tiab] OR Brazil[tiab] OR Bulgaria[tiab] OR Burkina Faso[tiab] OR Burkina Fasso[tiab] OR Upper Volta[tiab] OR Burundi[tiab] OR Urundi[tiab] OR Cambodia[tiab] OR Khmer Republic[tiab] OR Kampuchea[tiab] OR Cameroon[tiab] OR Cameroons[tiab] OR Cameron[tiab] OR Camerons[tiab] OR Cape Verde[tiab] OR Central African Republic[tiab] OR Chad[tiab] OR Chile[tiab] OR China[tiab] OR Colombia[tiab] OR Comoros[tiab] OR Comoro Islands[tiab] OR Comores[tiab] OR Mayotte[tiab] OR Congo[tiab] OR Zaire[tiab] OR Costa Rica[tiab] OR Cote d'Ivoire[tiab] OR Ivory Coast[tiab] OR Croatia[tiab] OR Cuba[tiab] OR Cyprus[tiab] OR Czechoslovakia[tiab] OR Czech Republic[tiab] OR Slovakia[tiab] OR Slovak Republic[tiab] OR Djibouti[tiab] OR French Somaliland[tiab] OR Dominica[tiab] OR Dominican Republic[tiab] OR East Timor[tiab] OR East Timur[tiab] OR Timor Leste[tiab] OR Ecuador[tiab] OR Egypt[tiab] OR United Arab Republic[tiab] OR El Salvador[tiab] OR Eritrea[tiab] OR Estonia[tiab] OR Ethiopia[tiab] OR Fiji[tiab] OR Gabon[tiab] OR Gabonese Republic[tiab] OR Gambia[tiab] OR Gaza[tiab] OR Georgia Republic[tiab] OR Georgian Republic[tiab] OR Ghana[tiab] OR Gold Coast[tiab] OR Greece[tiab] OR Grenada[tiab] OR Guatemala[tiab] OR Guinea[tiab] OR Guam[tiab] OR Guiana[tiab] OR Guyana[tiab] OR Haiti[tiab] OR Honduras[tiab] OR Hungary[tiab] OR India[tiab] OR Maldives[tiab] OR Indonesia[tiab] OR Iran[tiab] OR Iraq[tiab] OR Isle of Man[tiab] OR Jamaica[tiab] OR Jordan[tiab] OR Kazakhstan[tiab] OR Kazakh[tiab] OR Kenya[tiab] OR Kiribati[tiab] OR Korea[tiab] OR Kosovo[tiab] OR Kyrgyzstan[tiab] OR Kirghizia[tiab] OR Kyrgyz Republic[tiab] OR Kirghiz[tiab] OR Kirgizstan[tiab] OR "Lao PDR"[tiab] OR Laos[tiab] OR Latvia[tiab] OR Lebanon[tiab] OR Lesotho[tiab] OR Basutoland[tiab] OR Liberia[tiab] OR Libya[tiab] OR Lithuania[tiab])) OR (Macedonia[pl] OR Madagascar[pl] OR Malagasy Republic[pl] OR Malaysia[pl] OR Malaya[pl] OR Malay[pl] OR Sabah[pl] OR Sarawak[pl] OR Malawi[pl] OR Nyasaland[pl] OR Mali[pl] OR Malta[pl] OR Marshall Islands[pl] OR Mauritania[pl] OR Mauritius[pl] OR Agalega Islands[pl] OR Mexico[pl] OR Micronesia[pl] OR Middle East[pl] OR Moldova[pl] OR Moldovia[pl] OR Moldovian[pl] OR Mongolia[pl] OR Montenegro[pl] OR Morocco[pl] OR Ifni[pl] OR Mozambique[pl] OR Myanmar[pl] OR Myanma[pl] OR Burma[pl] OR Namibia[pl] OR Nepal[pl] OR Netherlands Antilles[pl] OR New Caledonia[pl] OR Nicaragua[pl] OR Niger[pl] OR Nigeria[pl] OR Northern Mariana Islands[pl] OR Oman[pl] OR Muscat[pl] OR Pakistan[pl] OR Palau[pl] OR Palestine[pl] OR Panama[pl] OR Paraguay[pl] OR Peru[pl] OR Philippines[pl] OR Philipines[pl] OR Phillipines[pl] OR Phillippines[pl] OR Poland[pl] OR Portugal[pl] OR Puerto Rico[pl] OR Romania[pl] OR Rumania[pl] OR Roumania[pl] OR Russia[pl] OR Russian[pl] OR Rwanda[pl] OR Ruanda[pl] OR Saint Kitts[pl] OR St Kitts[pl] OR Nevis[pl] OR Saint Lucia[pl] OR St Lucia[pl] OR Saint Vincent[pl] OR St Vincent[pl] OR Grenadines[pl] OR Samoa[pl] OR Samoan Islands[pl] OR Navigator Island[pl] OR Navigator Islands[pl] OR Sao Tome[pl] OR Saudi Arabia[pl] OR Senegal[pl] OR Serbia[pl] OR Montenegro[pl] OR Seychelles[pl] OR Sierra Leone[pl] OR Slovenia[pl] OR Sri Lanka[pl] OR Ceylon[pl] OR Solomon Islands[pl] OR Somalia[pl] OR South Africa[pl] OR Sudan[pl] OR Suriname[pl] OR Surinam[pl] OR Swaziland[pl] OR Syria[pl] OR Tajikistan[pl] OR Tadzhikistan[pl] OR Tadjikistan[pl] OR Tadzhik[pl] OR Tanzania[pl] OR Thailand[pl] OR Togo[pl] OR Togolese Republic[pl] OR Tonga[pl] OR Trinidad[pl] OR Tobago[pl] OR Tunisia[pl] OR Turkey[pl] OR Turkmenistan[pl] OR Turkmen[pl] OR Uganda[pl] OR Ukraine[pl] OR Uruguay[pl] OR USSR[pl] OR Soviet Union[pl] OR Union of Soviet Socialist Republics[pl] OR Uzbekistan[pl] OR Uzbek OR Vanuatu[pl] OR New Hebrides[pl] OR Venezuela[pl] OR Vietnam[pl] OR Viet Nam[pl] OR West Bank[pl] OR Yemen[pl] OR Yugoslavia[pl] OR Zambia[pl] OR Zimbabwe[pl] OR Rhodesia[pl])) OR (Africa[pl] OR Asia[pl] OR Caribbean[pl] OR West Indies[pl] OR South America[pl] OR Latin America[pl] OR Central America[pl] OR Afghanistan[pl] OR Albania[pl] OR Algeria[pl] OR Angola[pl] OR Antigua[pl] OR Barbuda[pl] OR Argentina[pl] OR Armenia[pl] OR Armenian[pl] OR Aruba[pl] OR Azerbaijan[pl] OR Bahrain[pl] OR Bangladesh[pl] OR Barbados[pl] OR Benin[pl] OR Byelarus[pl] OR Byelorussian[pl] OR Belarus[pl] OR Belorussian[pl] OR Belorussia[pl] OR Belize[pl] OR Bhutan[pl] OR Bolivia[pl] OR Bosnia[pl] OR Herzegovina[pl] OR Hercegovina[pl] OR Botswana[pl] OR Brasil[pl] OR Brazil[pl] OR Bulgaria[pl] OR Burkina Faso[pl] OR Burkina Fasso[pl] OR Upper Volta[pl] OR Burundi[pl] OR Urundi[pl] OR Cambodia[pl] OR Khmer Republic[pl] OR Kampuchea[pl] OR Cameroon[pl] OR Cameroons[pl] OR Cameron[pl] OR Camerons[pl] OR Cape Verde[pl] OR Central African Republic[pl] OR Chad[pl] OR Chile[pl] OR China[pl] OR Colombia[pl] OR Comoros[pl] OR Comoro Islands[pl] OR Comores[pl] OR Mayotte[pl] OR Congo[pl] OR Zaire[pl] OR Costa Rica[pl] OR Cote d'Ivoire[pl] OR Ivory Coast[pl] OR Croatia[pl] OR Cuba[pl] OR Cyprus[pl] OR Czechoslovakia[pl] OR Czech Republic[pl] OR Slovakia[pl] OR Slovak Republic[pl] OR Djibouti[pl] OR French Somaliland[pl] OR Dominica[pl] OR Dominican Republic[pl] OR East Timor[pl] OR East Timur[pl] OR Timor Leste[pl] OR Ecuador[pl] OR Egypt[pl] OR United Arab Republic[pl] OR El Salvador[pl] OR Eritrea[pl] OR Estonia[pl] OR Ethiopia[pl] OR Fiji[pl] OR Gabon[pl] OR Gabonese Republic[pl] OR Gambia[pl] OR Gaza[pl] OR Georgia Republic[pl] OR Georgian Republic[pl] OR Ghana[pl] OR Gold Coast[pl] OR Greece[pl] OR Grenada[pl] OR Guatemala[pl] OR Guinea[pl] OR Guam[pl] OR Guiana[pl] OR Guyana[pl] OR Haiti[pl] OR Honduras[pl] OR Hungary[pl] OR India[pl] OR Maldives[pl] OR Indonesia[pl] OR Iran[pl] OR Iraq[pl] OR Isle of Man[pl] OR Jamaica[pl] OR Jordan[pl] OR Kazakhstan[pl] OR Kazakh[pl] OR Kenya[pl] OR Kiribati[pl] OR Korea[pl] OR Kosovo[pl] OR Kyrgyzstan[pl] OR Kirghizia[pl] OR Kyrgyz Republic[pl] OR Kirghiz[pl] OR Kirgizstan[pl] OR "Lao PDR"[pl] OR Laos[pl] OR Latvia[pl] OR Lebanon[pl] OR Lesotho[pl] OR Basutoland[pl] OR Liberia[pl] OR Libya[pl] OR Lithuania[pl])) OR ("developing country"[ot] OR "developing countries"[ot] OR "developing nation"[ot] OR "developing nations"[ot] OR "developing population"[ot] OR "developing populations"[ot] OR "developing world"[ot] OR "less developed country"[ot] OR "less developed countries"[ot] OR "less developed nation"[ot] OR "less developed nations"[ot] OR "less developed population"[ot] OR "less developed populations"[ot] OR "less developed world"[ot] OR "lesser developed country"[ot] OR "lesser developed countries"[ot] OR "lesser developed nation"[ot] OR "lesser developed nations"[ot] OR "lesser developed population"[ot] OR "lesser developed populations"[ot] OR "lesser developed world"[ot] OR "under developed country"[ot] OR "under developed countries"[ot] OR "under developed nation"[ot] OR "under developed nations"[ot] OR "under developed population"[ot] OR "under developed populations"[ot] OR "under developed world"[ot] OR "underdeveloped country"[ot] OR "underdeveloped countries"[ot] OR "underdeveloped nation"[ot] OR "underdeveloped nations"[ot] OR "underdeveloped population"[ot] OR "underdeveloped populations"[ot] OR "underdeveloped world"[ot] OR "middle income country"[ot] OR "middle income countries"[ot] OR "middle income nation"[ot] OR "middle income nations"[ot] OR "middle income population"[ot] OR "middle income populations"[ot] OR "low income country"[ot] OR "low income countries"[ot] OR "low income nation"[ot] OR "low income nations"[ot] OR "low income population"[ot] OR "low income populations"[ot] OR "lower income country"[ot] OR "lower income countries"[ot] OR "lower income nation"[ot] OR "lower income nations"[ot] OR "lower income population"[ot] OR "lower income populations"[ot] OR "underserved country"[ot] OR "underserved countries"[ot] OR "underserved nation"[ot] OR "underserved nations"[ot] OR "underserved population"[ot] OR "underserved populations"[ot] OR "underserved world"[ot] OR "under served country"[ot] OR "under served countries"[ot] OR "under served nation"[ot] OR "under served nations"[ot] OR "under served population"[ot] OR "under served populations"[ot] OR "under served world"[ot] OR "deprived country"[ot] OR "deprived countries"[ot] OR "deprived nation"[ot] OR "deprived nations"[ot] OR "deprived population"[ot] OR "deprived populations"[ot] OR "deprived world"[ot] OR "poor country"[ot] OR "poor countries"[ot] OR "poor nation"[ot] OR "poor nations"[ot] OR "poor population"[ot] OR "poor populations"[ot] OR "poor world"[ot] OR "poorer country"[ot] OR "poorer countries"[ot] OR "poorer nation"[ot] OR "poorer nations"[ot] OR "poorer population"[ot] OR "poorer populations"[ot] OR "poorer world"[ot] OR "developing economy"[ot] OR "developing economies"[ot] OR "less developed economy"[ot] OR "less developed economies"[ot] OR "lesser developed economy"[ot] OR "lesser developed economies"[ot] OR "under developed economy"[ot] OR "under developed economies"[ot] OR "underdeveloped economy"[ot] OR "underdeveloped economies"[ot] OR "middle income economy"[ot] OR "middle income economies"[ot] OR "low income economy"[ot] OR "low income economies"[ot] OR "lower income economy"[ot] OR "lower income economies"[ot] OR "low gdp"[ot] OR "low gnp"[ot] OR "low gross domestic"[ot] OR "low gross national"[ot] OR "lower gdp"[ot] OR "lower gnp"[ot] OR "lower gross domestic"[ot] OR "lower gross national"[ot] OR lmic[ot] OR lmics[ot] OR "third world"[ot] OR "lami country"[ot] OR "lami countries"[ot] OR "transitional country"[ot] OR "transitional countries"[ot])) OR ("developing country"[tiab] OR "developing countries"[tiab] OR "developing nation"[tiab] OR "developing nations"[tiab] OR "developing population"[tiab] OR "developing populations"[tiab] OR "developing world"[tiab] OR "less developed country"[tiab] OR "less developed countries"[tiab] OR "less developed nation"[tiab] OR "less developed nations"[tiab] OR "less developed population"[tiab] OR "less developed populations"[tiab] OR "less developed world"[tiab] OR "lesser developed country"[tiab] OR "lesser developed countries"[tiab] OR "lesser developed nation"[tiab] OR "lesser developed nations"[tiab] OR "lesser developed population"[tiab] OR "lesser developed populations"[tiab] OR "lesser developed world"[tiab] OR "under developed country"[tiab] OR "under developed countries"[tiab] OR "under developed nation"[tiab] OR "under developed nations"[tiab] OR "under developed population"[tiab] OR "under developed populations"[tiab] OR "under developed world"[tiab] OR "underdeveloped country"[tiab] OR "underdeveloped countries"[tiab] OR "underdeveloped nation"[tiab] OR "underdeveloped nations"[tiab] OR "underdeveloped population"[tiab] OR "underdeveloped populations"[tiab] OR "underdeveloped world"[tiab] OR "middle income country"[tiab] OR "middle income countries"[tiab] OR "middle income nation"[tiab] OR "middle income nations"[tiab] OR "middle income population"[tiab] OR "middle income populations"[tiab] OR "low income country"[tiab] OR "low income countries"[tiab] OR "low income nation"[tiab] OR "low income nations"[tiab] OR "low income population"[tiab] OR "low income populations"[tiab] OR "lower income country"[tiab] OR "lower income countries"[tiab] OR "lower income nation"[tiab] OR "lower income nations"[tiab] OR "lower income population"[tiab] OR "lower income populations"[tiab] OR "underserved country"[tiab] OR "underserved countries"[tiab] OR "underserved nation"[tiab] OR "underserved nations"[tiab] OR "underserved population"[tiab] OR "underserved populations"[tiab] OR "underserved world"[tiab] OR "under served country"[tiab] OR "under served countries"[tiab] OR "under served nation"[tiab] OR "under served nations"[tiab] OR "under served population"[tiab] OR "under served populations"[tiab] OR "under served world"[tiab] OR "deprived country"[tiab] OR "deprived countries"[tiab] OR "deprived nation"[tiab] OR "deprived nations"[tiab] OR "deprived population"[tiab] OR "deprived populations"[tiab] OR "deprived world"[tiab] OR "poor country"[tiab] OR "poor countries"[tiab] OR "poor nation"[tiab] OR "poor nations"[tiab] OR "poor population"[tiab] OR "poor populations"[tiab] OR "poor world"[tiab] OR "poorer country"[tiab] OR "poorer countries"[tiab] OR "poorer nation"[tiab] OR "poorer nations"[tiab] OR "poorer population"[tiab] OR "poorer populations"[tiab] OR "poorer world"[tiab] OR "developing economy"[tiab] OR "developing economies"[tiab] OR "less developed economy"[tiab] OR "less developed economies"[tiab] OR "lesser developed economy"[tiab] OR "lesser developed economies"[tiab] OR "under developed economy"[tiab] OR "under developed economies"[tiab] OR "underdeveloped economy"[tiab] OR "underdeveloped economies"[tiab] OR "middle income economy"[tiab] OR "middle income economies"[tiab] OR "low income economy"[tiab] OR "low income economies"[tiab] OR "lower income economy"[tiab] OR "lower income economies"[tiab] OR "low gdp"[tiab] OR "low gnp"[tiab] OR "low gross domestic"[tiab] OR "low gross national"[tiab] OR "lower gdp"[tiab] OR "lower gnp"[tiab] OR "lower gross domestic"[tiab] OR "lower gross national"[tiab] OR lmic[tiab] OR lmics[tiab] OR "third world"[tiab] OR "lami country"[tiab] OR "lami countries"[tiab] OR "transitional country"[tiab] OR "transitional countries"[tiab]) |
| 4 | 1 AND 2 AND 3 AND Filter: Publication date from 2000/01/01 to 2015/12/31 | |
