# Supplementary material for: Scoping review to identify and map the health personnel considered skilled birth attendants in low-and-middle income countries from 2000–2015
Source: PLoS One. 2019 Feb 1;14(2):e0211576. doi: 10.1371/journal.pone.0211576 (PMC6358074; doi:10.1371/journal.pone.0211576)
Supplement: S1 Table — (DOCX) [file pone.0211576.s003.docx]

| **Ref. ID** | **First Author** | **Year of Publication** | **Year(s) of Study Coverage** | **Study Design** | **Country** | **Level of Represen-tation** | **Study Location** | **Study Setting** | **Cadre Name** | **Skilled** | **Parenteral Antibiotics** | **Parenteral Uterotonic Drugs** | **Parenteral Anti-convulsants** | **Manually Removal of Placenta** | **Remove Retained Products** | **Assisted Vaginal Delivery** | **Neonatal Resuscitation** | **Surgery (i.e. C-Section)** | **Blood Transfusion** | **Education Detail** |
| --- | --- | --- | --- | --- | --- | --- | --- | --- | --- | --- | --- | --- | --- | --- | --- | --- | --- | --- | --- | --- |
| [1] | Adegoke | 2012 | 2009-2011 | Cross-sectional | Gambia | National | Health Facility and Community | Rural and Urban | Community Health Nurse Midwife | Yes | Perform | Not performed | Not performed | Not performed | Not performed | Not performed | Perform | Not performed | Perform | Not stated |
|  |  |  |  |  |  |  |  |  | Enrolled Midwife | Yes | Perform | Perform | Perform | Perform | Perform | Perform | Perform | Perform | Perform | Not stated |
|  |  |  |  |  |  |  |  |  | Enrolled Nurse | Yes | Perform | Not performed | Not performed | Not performed | Not performed | Not performed | Perform | Not performed | Not performed | Not stated |
|  |  |  |  |  |  |  |  |  | Enrolled Nurse Midwife | Yes | Perform | Perform | Perform | Perform | Not performed | Not performed | Perform | Not performed | Perform | Not stated |
|  |  |  |  |  |  |  |  |  | General Doctor | Yes | Perform | Not performed | Not performed | Not performed | Not performed | Not performed | Perform | Not performed | Not performed | Not stated |
|  |  |  |  |  |  |  |  |  | Obstetrician / Gynecologist | Yes | Perform | Perform | Perform | Perform | Perform | Perform | Perform | Perform | Perform | Not stated |
|  |  |  |  |  |  |  |  |  | Registered General Nurse | Yes | Perform | Not performed | Not performed | Not performed | Not performed | Not performed | Perform | Not performed | Perform | Not stated |
|  |  |  |  |  |  |  |  |  | Registered Midwife | Yes | Perform | Perform | Perform | Perform | Perform | Perform | Perform | Not performed | Perform | Not stated |
|  |  |  |  |  |  |  |  |  | Registered Nurse Midwife | Yes | Perform | Perform | Perform | Perform | Perform | Perform | Perform | Not performed | Perform | Not stated |
|  |  |  |  |  | Ghana | National | Health Facility and Community | Rural and Urban | Auxiliary Midwife | Yes | Perform | Perform | Not performed | Not performed | Not performed | Not performed | Not performed | Not performed | Not performed | Not stated |
|  |  |  |  |  |  |  |  |  | Community Health Nurse Midwife | Yes | Perform | Perform | Perform | Perform | Perform | Perform | Perform | Not performed | Perform | Not stated |
|  |  |  |  |  |  |  |  |  | Enrolled Nurse Midwife | Yes | Perform | Perform | Perform | Perform | Perform | Perform | Perform | Not performed | Perform | Not stated |
|  |  |  |  |  |  |  |  |  | General Doctor | Yes | Perform | Perform | Perform | Perform | Perform | Perform | Perform | Perform | Perform | Not stated |
|  |  |  |  |  |  |  |  |  | Medical Assistant | Yes | Perform | Perform | Perform | Perform | Perform | Perform | Perform | Not performed | Perform | Not stated |
|  |  |  |  |  |  |  |  |  | Obstetrician / Gynecologist | Yes | Perform | Perform | Perform | Perform | Perform | Perform | Perform | Perform | Perform | Not stated |
|  |  |  |  |  |  |  |  |  | Registered Midwife | Yes | Perform | Perform | Perform | Perform | Perform | Perform | Perform | Not performed | Perform | Not stated |
|  |  |  |  |  |  |  |  |  | Registered Nurse Midwife | Yes | Perform | Perform | Perform | Perform | Perform | Perform | Perform | Not performed | Perform | Not stated |
|  |  |  |  |  | Kenya | National | Health Facility and Community | Rural and Urban | Clinical Officer | Yes | Perform | Perform | Perform | Not performed | Perform | Not performed | Perform | Not performed | Perform | Not stated |
|  |  |  |  |  |  |  |  |  | Community Midwife | Yes | Perform | Perform | Perform | Perform | Not performed | Not performed | Perform | Not performed | Not performed | Not stated |
|  |  |  |  |  |  |  |  |  | Enrolled Midwife | Yes | Perform | Perform | Perform | Perform | Perform | Perform | Perform | Not performed | Perform | Not stated |
|  |  |  |  |  |  |  |  |  | Enrolled Nurse | Yes | Perform | Perform | Perform | Perform | Perform | Perform | Perform | Not performed | Perform | Not stated |
|  |  |  |  |  |  |  |  |  | Enrolled Nurse Midwife | No consensus | Perform | Perform | Perform | Perform | Perform | Not performed | Perform | Perform | Not stated | Not stated |
|  |  |  |  |  |  |  |  |  | General Doctor | Yes | Perform | Perform | Perform | Perform | Perform | Perform | Perform | Perform | Perform | Not stated |
|  |  |  |  |  |  |  |  |  | Obstetrician / Gynecologist | Yes | Perform | Perform | Perform | Perform | Perform | Perform | Perform | Perform | Perform | Not stated |
|  |  |  |  |  |  |  |  |  | Registered Community Health Nurse | Yes | Perform | Perform | Perform | Perform | Perform | Perform | Perform | Not performed | Perform | Not stated |
|  |  |  |  |  |  |  |  |  | Registered General Nurse | Yes | Perform | Perform | Perform | Perform | Perform | Perform | Perform | Not performed | Perform | Not stated |
|  |  |  |  |  |  |  |  |  | Registered Nurse Midwife | Yes | Perform | Perform | Perform | Perform | Perform | Perform | Perform | Not performed | Perform | Not stated |
|  |  |  |  |  | Malawi | National | Health Facility and Community | Rural and Urban | Clinical Officer | Yes | Perform | Perform | Perform | Perform | Perform | Perform | Perform | Perform | Perform | Not stated |
|  |  |  |  |  |  |  |  |  | Enrolled Midwife | Yes | Perform | Perform | Perform | Perform | Perform | Perform | Perform | Not performed | Perform | Not stated |
|  |  |  |  |  |  |  |  |  | Enrolled Nurse | Yes | Perform | Not performed | Not performed | Not performed | Not performed | Not performed | Not performed | Not performed | Perform | Not stated |
|  |  |  |  |  |  |  |  |  | Enrolled Nurse Midwife | Yes | Perform | Perform | Perform | Perform | Perform | Perform | Perform | Not performed | Perform | Not stated |
|  |  |  |  |  |  |  |  |  | General Doctor | Yes | Perform | Perform | Perform | Perform | Perform | Perform | Perform | Perform | Perform | Not stated |
|  |  |  |  |  |  |  |  |  | Maternal and Child Health Aide | Yes | Perform | Not performed | Not performed | Not performed | Not performed | Not performed | Not performed | Not performed | Not performed | Not stated |
|  |  |  |  |  |  |  |  |  | Medical Assistant | Yes | Perform | Perform | Perform | Perform | Perform | Perform | Perform | Not performed | Perform | Not stated |
|  |  |  |  |  |  |  |  |  | Nurse Midwife Technician | Yes | Perform | Perform | Perform | Perform | Perform | Perform | Perform | Not performed | Perform | Not stated |
|  |  |  |  |  |  |  |  |  | Nurse Technician | Yes | Perform | Not performed | Not performed | Not performed | Not performed | Not performed | Not performed | Not performed | Perform | Not stated |
|  |  |  |  |  |  |  |  |  | Obstetrician / Gynecologist | Yes | Perform | Perform | Perform | Perform | Perform | Perform | Perform | Perform | Perform | Not stated |
|  |  |  |  |  |  |  |  |  | Registered Nurse Midwife | Yes | Perform | Perform | Perform | Perform | Perform | Perform | Perform | Not performed | Perform | Not stated |
|  |  |  |  |  | Nigeria | National | Health Facility and Community | Rural and Urban | Community Health Extension Worker | No | Perform | Perform | Perform | Perform | Not performed | Not performed | Not performed | Not performed | Not performed | Not stated |
|  |  |  |  |  |  |  |  |  | General Doctor | Yes | Perform | Perform | Perform | Perform | Perform | Perform | Perform | Perform | Perform | Not stated |
|  |  |  |  |  |  |  |  |  | Junior Community Heatlh Extension Worker | No | Perform | Perform | Perform | Perform | Not performed | Not performed | Not performed | Not performed | Not performed | Not stated |
|  |  |  |  |  |  |  |  |  | Obstetrician / Gynecologist | Yes | Perform | Perform | Perform | Perform | Perform | Perform | Perform | Perform | Perform | Not stated |
|  |  |  |  |  |  |  |  |  | Registered Midwife | Yes | Perform | Perform | Perform | Perform | Not performed | Not performed | Perform | Not performed | Perform | Not stated |
|  |  |  |  |  |  |  |  |  | Registered Nurse Midwife | Yes | Perform | Perform | Perform | Not performed | Not performed | Not performed | Perform | Not performed | Perform | Not stated |
|  |  |  |  |  | Sierra Leone | National | Health Facility and Community | Rural and Urban | Clinical Officer | Yes | Perform | Perform | Perform | Not performed | Not performed | Not performed | Perform | Not performed | Perform | Not stated |
|  |  |  |  |  |  |  |  |  | Enrolled Nurse Midwife | Yes | Perform | Perform | Perform | Perform | Not performed | Not performed | Perform | Not performed | Not performed | Not stated |
|  |  |  |  |  |  |  |  |  | General Doctor | Yes | Perform | Perform | Perform | Not performed | Perform | Not performed | Perform | Perform | Perform | Not stated |
|  |  |  |  |  |  |  |  |  | Maternal and Child Health Aide | No consensus | Perform | Perform | Perform | Not performed | Not performed | Not performed | Perform | Not performed | Not performed | Not stated |
|  |  |  |  |  |  |  |  |  | Obstetrician / Gynecologist | Yes | Perform | Perform | Perform | Not performed | Perform | Not performed | Perform | Perform | Perform | Not stated |
|  |  |  |  |  |  |  |  |  | Registered Midwife | Yes | Perform | Perform | Perform | Perform | Perform | Perform | Perform | Not performed | Perform | Not stated |
|  |  |  |  |  |  |  |  |  | Registered Nurse Midwife | Yes | Perform | Perform | Perform | Perform | Perform | Perform | Perform | Not performed | Perform | Not stated |
|  |  |  |  |  |  |  |  |  | State Enrolled Community Health Nurse | Yes | Perform | Perform | Perform | Not performed | Not performed | Not performed | Not performed | Not performed | Not performed | Not stated |
|  |  |  |  |  | Somalia | National | Health Facility and Community | Rural and Urban | Auxiliary Midwife | No consensus | Perform | Not performed | Not performed | Not performed | Not performed | Not performed | Not performed | Not performed | Not performed | Not stated |
|  |  |  |  |  |  |  |  |  | Clinical Officer | Yes | Perform | Perform | Perform | Not performed | Not performed | Not performed | Perform | Not performed | Perform | Not stated |
|  |  |  |  |  |  |  |  |  | Community Midwife | Yes | Perform | Perform | Perform | Perform | Not performed | Not performed | Perform | Not performed | Perform | Not stated |
|  |  |  |  |  |  |  |  |  | General Doctor | Yes | Perform | Perform | Perform | Perform | Perform | Perform | Perform | Not performed | Perform | Not stated |
|  |  |  |  |  |  |  |  |  | Nurse Midwife Technician | Yes | Perform | Perform | Perform | Perform | Not performed | Not performed | Perform | Not performed | Perform | Not stated |
|  |  |  |  |  |  |  |  |  | Obstetrician / Gynecologist | Yes | Perform | Perform | Perform | Perform | Perform | Perform | Perform | Not performed | Perform | Not stated |
|  |  |  |  |  |  |  |  |  | Registered General Nurse | Yes | Perform | Perform | Perform | Perform | Perform | Perform | Perform | Not performed | Perform | Not stated |
|  |  |  |  |  |  |  |  |  | Registered Nurse Midwife | Yes | Perform | Perform | Perform | Perform | Perform | Perform | Perform | Not performed | Perform | Not stated |
|  |  |  |  |  | United Republic of Tanzania | National | Health Facility and Community | Rural and Urban | Assistant Clinical Officer | Yes | Perform | Perform | Perform | Perform | Not performed | Not performed | Perform | Not performed | Not performed | Not stated |
|  |  |  |  |  |  |  |  |  | Assistant Medical Officer | Yes | Perform | Perform | Perform | Perform | Perform | Not performed | Perform | Perform | Perform | Not stated |
|  |  |  |  |  |  |  |  |  | Enrolled Nurse | Yes | Perform | Perform | Perform | Perform | Not performed | Not performed | Perform | Not performed | Perform | Not stated |
|  |  |  |  |  |  |  |  |  | Enrolled Nurse Midwife | Yes | Perform | Perform | Perform | Perform | Perform | Not performed | Perform | Not performed | Perform | Not stated |
|  |  |  |  |  |  |  |  |  | General Doctor | Yes | Perform | Perform | Perform | Perform | Perform | Not performed | Perform | Perform | Perform | Not stated |
|  |  |  |  |  |  |  |  |  | Health Officer | Yes | Perform | Perform | Perform | Perform | Perform | Not performed | Perform | Not performed | Perform | Not stated |
|  |  |  |  |  |  |  |  |  | Maternal and Child Health Aide | Yes | Perform | Perform | Perform | Perform | Not performed | Not performed | Perform | Not performed | Not performed | Not stated |
|  |  |  |  |  |  |  |  |  | Obstetrician / Gynecologist | Yes | Perform | Perform | Perform | Perform | Perform | Not performed | Perform | Perform | Perform | Not stated |
|  |  |  |  |  |  |  |  |  | Registered Midwife | No consensus | Perform | Perform | Perform | Perform | Perform | Not performed | Perform | Not performed | Perform | Not stated |
|  |  |  |  |  |  |  |  |  | Registered Nurse Midwife | Yes | Perform | Perform | Perform | Perform | Perform | Not performed | Perform | Not performed | Perform | Not stated |
|  |  |  |  |  | Zimbabwe | National | Health Facility and Community | Rural and Urban | Clinical Officer | Yes | Perform | Perform | Perform | Not performed | Perform | Not performed | Perform | Perform | Perform | Not stated |
|  |  |  |  |  |  |  |  |  | General Doctor | Yes | Perform | Perform | Perform | Not performed | Not performed | Not performed | Perform | Perform | Perform | Not stated |
|  |  |  |  |  |  |  |  |  | Obstetrician / Gynecologist | Yes | Perform | Perform | Perform | Not performed | Not performed | Not performed | Perform | Perform | Perform | Not stated |
|  |  |  |  |  |  |  |  |  | Registered General Nurse | Yes | Perform | Perform | Not performed | Not performed | Not performed | Not performed | Perform | Not performed | Perform | Not stated |
|  |  |  |  |  |  |  |  |  | Registered Nurse Midwife | Yes | Perform | Perform | Perform | Perform | Not performed | Not performed | Perform | Not performed | Perform | Not stated |
|  |  |  |  |  |  |  |  |  | State Certified Nurse Midwife | Yes | Perform | Perform | Not performed | Not performed | Not performed | Not performed | Perform | Not performed | Perform | Not stated |
| [2] | Adegoke | 2013 | Not stated | Cross-sectional | Nigeria | States (n=3) in Northern Nigeria | Health Clinic and Community | Rural and Urban | Community Health Extension Worker | Yes | Not stated | Not stated | Not stated | Not stated | Not stated | Not stated | Not stated | Not stated | Not stated | No formal education |
|  |  |  |  |  |  |  |  |  | Junior Community Heatlh Extension Worker | Yes | Not stated | Not stated | Not stated | Not stated | Not stated | Not stated | Not stated | Not stated | Not stated | No formal education |
|  |  |  |  |  |  |  |  |  | Midwife | Yes | Not stated | Not stated | Not stated | Not stated | Not stated | Not stated | Not stated | Not stated | Not stated | Secondary school + 3 years training |
|  |  |  |  |  |  |  |  |  | Nurse | Yes | Not stated | Not stated | Not stated | Not stated | Not stated | Not stated | Not stated | Not stated | Not stated | Secondary school + 3 years training |
| [3] | Anderson | 2014 | 1989-2010 | Mixed methods | Ghana | All eligible graduates from Ghanian university based certified OB/GYN postgraduate training programs (n=2) | Health Facility | Rural and Urban | Obstetrician / Gynecologist | Yes | Not stated | Not stated | Not stated | Not stated | Not stated | Not stated | Not stated | Not stated | Not stated | Degree + residency training |
|  |  |  |  |  |  |  |  |  | Obstetrician / Gynecologist | Yes | Not stated | Not stated | Not stated | Not stated | Not stated | Not stated | Not stated | Not stated | Not stated | Degree + residency training |
| [4] | Ariff | 2010 | Not stated | Cross-sectional | Pakistan | National | Health Facility (Secondary and Tertiary) | Rural and Urban | Lady Health Visitor | Not stated | Not stated | Not stated | Not stated | Not stated | Not stated | Not stated | Not stated | Not stated | Not stated | Degree + 1 year internship |
|  |  |  |  |  |  |  | Community (Primary Care) | Rural and Urban | Lady Health Worker | Not stated | Not stated | Not stated | Not stated | Not stated | Not stated | Not stated | Some perform | Not stated | Not stated | Not stated |
|  |  |  |  |  |  |  | Hospital (Secondary and Tertiary) | Rural and Urban | Medical Officer | Not stated | Not stated | Not stated | Not stated | Not stated | Not stated | Not stated | Some perform | Not stated | Not stated | Not stated |
|  |  |  |  |  |  |  | Health Facility (Secondary and Tertiary) | Rural and Urban | Midwife | Not stated | Not stated | Not stated | Not stated | Not stated | Not stated | Not stated | Not stated | Not stated | Not stated | No formal education |
| [5] | Ayiasi | 2014 | 2011 | Cross-sectional | Uganda | District (n=1) | Health Facility | Rural | Midwife | Not stated | Not stated | Not stated | Not stated | Not stated | Not stated | Not stated | Not stated | Not stated | Not stated | No formal education |
|  |  |  |  |  |  |  |  |  | Nurse | Not stated | Not stated | Not stated | Not stated | Not stated | Not stated | Not stated | Not stated | Not stated | Not stated | Not stated |
|  |  |  |  |  |  |  |  |  | Nursing Assistant | Not stated | Not stated | Not stated | Not stated | Not stated | Not stated | Not stated | Not stated | Not stated | Not stated | Not stated |
| [6] | Bharati | 2015 | 2013 | Cross-sectional | India | State (n=1) | Health Facility | Urban | Student Nurse | Yes | Not stated | Not stated | Not stated | Not stated | Not stated | Not stated | Not stated | Not stated | Not stated | Diploma in nursing |
| [7] | Bhuiyan | 2005 | 2003 | Mixed methods | Bangladesh | National | Health Facility | Rural and Urban | Family Welfare Assistant | No | Not stated | Not stated | Not stated | Not stated | Not stated | Not stated | Not stated | Not stated | Not stated | Not stated |
|  |  |  |  |  |  |  |  |  | Female Health Assistant | No | Not stated | Not stated | Not stated | Not stated | Not stated | Not stated | Not stated | Not stated | Not stated | Not stated |
| [8] | Bogren | 2013 | Not stated | Mixed methods | Nepal | Key stakeholders from Government of Nepal, professional organizations, NGO's, UN agencies and bi-lateral organizations | Health Facility | Rural and Urban | Auxiliary Nurse Midwife | No | Not stated | Not stated | Not stated | Not stated | Not stated | Not stated | Not stated | Not stated | Not stated | Secondary school + 1.5 years training |
|  |  |  |  |  |  |  |  |  | Nurse (BN) | No | Not stated | Not stated | Not stated | Not stated | Not stated | Not stated | Not stated | Not stated | Not stated | Auxiliary Nurse + 2 months + 252 practice hours |
|  |  |  |  |  |  |  |  |  | Nurse (Generic BSc in Nursing) | No | Not stated | Not stated | Not stated | Not stated | Not stated | Not stated | Not stated | Not stated | Not stated | Degree |
|  |  |  |  |  |  |  |  |  | Nurse (Proficiency Certificate Level) | No | Not stated | Not stated | Not stated | Not stated | Not stated | Not stated | Not stated | Not stated | Not stated | Degree |
|  |  |  |  |  |  |  |  |  | Professional Midwife | No | Not stated | Not stated | Not stated | Not stated | Not stated | Not stated | Not stated | Not stated | Not stated | Certificate |
| [9] | Bradley | 2009 | Not stated | Qualitative | Malawi | Hospitals (n=4) | Hospital | Rural | Clinical Officer | Not stated | Not stated | Not stated | Not stated | Not stated | Not stated | Not stated | Not stated | Not stated | Not stated | Not stated |
|  |  |  |  |  |  |  |  |  | Enrolled Nurse Midwife | Not stated | Not stated | Not stated | Not stated | Not stated | Not stated | Not stated | Not stated | Not stated | Not stated | Not stated |
|  |  |  |  |  |  |  |  |  | Medical Assistant | Not stated | Not stated | Not stated | Not stated | Not stated | Not stated | Not stated | Not stated | Not stated | Not stated | Not stated |
|  |  |  |  |  |  |  |  |  | Nurse Midwife Technician | Not stated | Not stated | Not stated | Not stated | Not stated | Not stated | Not stated | Not stated | Not stated | Not stated | Not stated |
|  |  |  |  |  |  |  |  |  | Registered Nurse Midwife | Not stated | Not stated | Not stated | Not stated | Not stated | Not stated | Not stated | Not stated | Not stated | Not stated | Not stated |
| [10] | Carlo | 2009 | 2005 | Cohort | Zambia | City (n=2) | Health Clinic | Urban | Nurse Midwife | Yes | Not stated | Not stated | Not stated | Not stated | Not stated | Not stated | Some perform | Not stated | Not stated | Not stated |
| [11] | Carlough | 2005 | 2005 | Qualitative | Nepal | Districts (n=4) | Community | Rural and Urban | Maternal and Child Health Worker | Yes | Perform | Not stated | Perform | Not stated | Not stated | Not stated | Not stated | Not stated | Not stated | Secondary school + 15 week course |
| [12] | Cheung | 2011 | Not stated | Mixed methods | China | Hospitals (n=10) in n=7 cities | Hospital | Urban | Midwife | Yes | Not stated | Not stated | Not stated | Not stated | Not stated | Not stated | Not stated | Not stated | Not stated | Degree |
| [13] | Danda | 2015 | 2014 | Cross-sectional | Zimbabwe | Hospitals (n=2) | Hospital | Urban | Midwife | Yes | Not stated | Not stated | Not stated | Not stated | Not stated | Not stated | Not stated | Not stated | Not stated | Not stated |
| [14] | Danishevski | 2009 | 2004-2005 | Mixed methods | Russia | Health facilities (n=10 public, n=2 faith based) in Districts (n=2). | Health Facility | Not stated | Nurse | Yes | Not stated | Not stated | Not stated | Not stated | Not stated | Not stated | Not stated | Not stated | Not stated | Degree + residency training |
|  |  |  |  |  |  |  |  |  | Obstetrician / Gynecologist | Yes | Not stated | Not stated | Not stated | Not stated | Not stated | Not stated | Not stated | Not stated | Not stated | Degree |
| [15] | Dawson | 2015 | 2011-2012 | Mixed methods | Papua New Guinea | Hospitals (n=4) with midwifery schools | Hospital | Not stated | Midwife | Yes | Not stated | Not stated | Not stated | Not stated | Not stated | Not stated | Not stated | Not stated | Not stated | Midwifery school, 12 months |
| [16] | del Hierro | 2014 | Not stated | Cross-sectional | Ecuador | Province (n=1) | Health Center | Rural | Doctor | Yes | Not stated | Not stated | Not stated | Not stated | Not stated | Not stated | Not stated | Not stated | Not stated | Degree + residency training |
| [17] | DeMaria | 2012 | 2007 | Mixed methods | Mexico | Hospitals (n=2) | Hospital | Urban | Generalist Physician | Yes | Not stated | Not stated | Not stated | Not stated | Not stated | Not stated | Not stated | Not performed | Not stated | Degree + residency training |
|  |  |  |  |  |  |  |  |  | Obstetric Nurse | Yes | Not stated | Not stated | Not stated | Not stated | Not stated | Not stated | Not stated | Not performed | Not stated | Degree + one yr in-service attending >20 births |
|  |  |  |  |  |  |  |  |  | Professional Midwife | Yes | Not stated | Not stated | Not stated | Not stated | Not stated | Not stated | Not stated | Not performed | Not stated | Degree |
| [18] | Dgedge | 2014 | 2008-2009 | Cross-sectional | Mozambique | National | Health Facility | Rural and Urban | General Nurse | Not stated | Not stated | Not stated | Not stated | Not stated | Not stated | Not stated | Not stated | Not stated | Not stated | No formal education |
|  |  |  |  |  |  |  |  |  | Maternal and Child Health Nurse (B-level) | Not stated | Not stated | Not stated | Not stated | Not stated | Not stated | Not stated | Not stated | Not stated | Not stated | No formal education |
|  |  |  |  |  |  |  |  |  | Maternal and Child Health Nurse (Mid-level) | Not stated | Not stated | Not stated | Not stated | Not stated | Not stated | Not stated | Not stated | Not stated | Not stated | Not stated |
|  |  |  |  |  |  |  |  |  | Medical Agent (Basic-level) | Not stated | Not stated | Not stated | Not stated | Not stated | Not stated | Not stated | Not stated | Not stated | Not stated | Not stated |
|  |  |  |  |  |  |  |  |  | Medical Technician (Mid-level) | Not stated | Not stated | Not stated | Not stated | Not stated | Not stated | Not stated | Not stated | Not stated | Not stated | Not stated |
|  |  |  |  |  |  |  |  |  | Nurse | Not stated | Not stated | Not stated | Not stated | Not stated | Not stated | Not stated | Not stated | Not stated | Not stated | Not stated |
| [19] | Dickerson | 2014 | Not stated | Mixed methods | Paraguay | Districts (n=5) | Hospital | Rural and Urban | Midwife | Yes | Not stated | Not stated | Not stated | Not stated | Not stated | Not stated | Not stated | Not stated | Not stated | Degree |
| [20] | Dogba | 2012 | Not stated | Qualitative | Mali | Community Health Centers (n=25) in one Region | Health Center (Community) | Rural | Doctor | Yes | Not stated | Not stated | Not stated | Not stated | Not stated | Not stated | Not stated | Not stated | Not stated | Not stated |
|  |  |  |  |  |  |  |  |  | Matron | Yes | Not stated | Not stated | Not stated | Not stated | Not stated | Not stated | Not stated | Not stated | Not stated | Degree |
|  |  |  |  |  |  |  |  |  | Midwife | Yes | Not stated | Not stated | Not stated | Not stated | Not stated | Not stated | Not stated | Not stated | Not stated | Not stated |
|  |  |  |  |  |  |  |  |  | Midwife | Yes | Not stated | Not stated | Not stated | Not stated | Not stated | Not stated | Not stated | Not stated | Not stated | Not stated |
|  |  |  |  |  |  |  |  |  | Nurse | Yes | Not stated | Not stated | Not stated | Not stated | Not stated | Not stated | Not stated | Not stated | Not stated | Diploma |
|  |  |  |  |  |  |  |  |  | Obstetric Nurse | Yes | Not stated | Not stated | Not stated | Not stated | Not stated | Not stated | Not stated | Not stated | Not stated | No formal education |
| [21] | Erlandsson | 2014 | 2013 | Qualitative | Nepal | Hospitals (n=2) | Hospital (Tertiary) | Not stated | Auxiliary Nurse Midwife | Not stated | Not stated | Not stated | Not stated | Not stated | Not stated | Not stated | Not stated | Not stated | Not stated | Not stated |
|  |  |  |  |  |  |  |  |  | Certified Nurse | Not stated | Not stated | Not stated | Not stated | Not stated | Not stated | Not stated | Not stated | Not stated | Not stated | Not stated |
|  |  |  |  |  |  |  |  |  | Doctor | Not stated | Not stated | Not stated | Not stated | Not stated | Not stated | Not stated | Not stated | Not stated | Not stated | Not stated |
|  |  |  |  |  |  |  |  |  | Nurse | Not stated | Not stated | Not stated | Not stated | Not stated | Not stated | Not stated | Not stated | Not stated | Not stated | Degree |
| [22] | Evans | 2014 | 2012 | Quasi-experimental | India | City (n=2) | Health Facility | Urban | Auxiliary Nurse Midwife | Semi-skilled | Not stated | Not stated | Not stated | Some perform | Not stated | Not stated | Not stated | Not stated | Not stated | Not stated |
|  |  |  |  |  |  |  |  |  | Doctor | Semi-skilled | Not stated | Not stated | Not stated | Perform | Not stated | Not stated | Not stated | Not stated | Not stated | Not stated |
|  |  |  |  |  |  |  |  |  | Nurse | Semi-skilled | Not stated | Not stated | Not stated | Some perform | Not stated | Not stated | Not stated | Not stated | Not stated | Not stated |
|  |  |  |  |  | Malawi | District (n=1) | Health Facility | Rural and Urban | Clinical Officer | Semi-skilled | Not stated | Not stated | Not stated | Some perform | Not stated | Not stated | Not stated | Not stated | Not stated | Not stated |
|  |  |  |  |  |  |  |  |  | Nurse Midwife | Semi-skilled | Not stated | Not stated | Not stated | Some perform | Not stated | Not stated | Not stated | Not stated | Not stated | Not stated |
|  |  |  |  |  | United Republic of Tanzania | Region (n=1) | Health Facility | Rural and Urban | Health Orderly | Not stated | Not stated | Not stated | Not stated | Not stated | Not stated | Not stated | Not stated | Not stated | Not stated | Not stated |
| [23] | Ezeonwu | 2011 | Not stated | Cross-sectional | Nigeria | State (n=1) | Health Facility | Rural and Urban | Antenatal Mother | Yes | Not stated | Not stated | Not stated | Not stated | Not stated | Not stated | Not stated | Not stated | Not stated | Not stated |
|  |  |  |  |  |  |  |  |  | Doctor | Yes | Not stated | Not stated | Not stated | Not stated | Not stated | Not stated | Not stated | Not stated | Not stated | Not stated |
|  |  |  |  |  |  |  |  |  | Midwife | Yes | Not stated | Not stated | Not stated | Not stated | Not stated | Not stated | Not stated | Not stated | Not stated | Not stated |
|  |  |  |  |  |  |  |  |  | Senior Nursing Sister | Yes | Not stated | Not stated | Not stated | Not stated | Not stated | Not stated | Not stated | Not stated | Not stated | Not stated |
|  |  |  |  |  |  |  |  |  | Staff Nurse | Yes | Not stated | Not stated | Not stated | Not stated | Not stated | Not stated | Not stated | Not stated | Not stated | Not stated |
|  |  |  |  |  |  |  |  |  | Staff Nurse | Yes | Not stated | Not stated | Not stated | Not stated | Not stated | Not stated | Not stated | Not stated | Not stated | Not stated |
|  |  |  |  |  |  |  |  |  | Ward Sister | Yes | Not stated | Not stated | Not stated | Not stated | Not stated | Not stated | Not stated | Not stated | Not stated | Not stated |
| [24] | Fawole | 2008 | Not stated | Cross-sectional | Nigeria | States (n=3) in Southwestern Nigeria | Health Facility (Public and Private) | Rural and Urban | Auxiliary Nurse | Not stated | Not stated | Not stated | Not stated | Not stated | Not stated | Not stated | Not stated | Not stated | Not stated | No formal education |
|  |  |  |  |  |  |  |  |  | Community Health Extension Worker | Not stated | Not stated | Not stated | Not stated | Not stated | Not stated | Not stated | Not stated | Not stated | Not stated | Not stated |
|  |  |  |  |  |  |  |  |  | Doctor | Not stated | Not stated | Not stated | Not stated | Not stated | Not stated | Not stated | Not stated | Not stated | Not stated | Not stated |
|  |  |  |  |  |  |  |  |  | Nurse Midwife | Not stated | Not stated | Not stated | Not stated | Not stated | Not stated | Not stated | Not stated | Not stated | Not stated | Not stated |
| [25] | Ferdous | 2011 | 2008 | Cross-sectional | Bangladesh | Hospitals (n=2) in Districts (n=2) | Hospital | Urban | Nurse | Yes | Not stated | Not stated | Not stated | Not performed | Not stated | Not stated | Not stated | Not stated | Not stated | Not stated |
| [26] | Fichardt | 2000 | Not stated | Cross-sectional | South Africa | Province (n=1) | Health Facility | Not stated | Registered Nurse | Not stated | Not stated | Not stated | Not stated | Not stated | Not stated | Not stated | Not stated | Not stated | Not stated | Not stated |
| [27] | Fikree | 2006 | 2003 | Cross-sectional | Pakistan | District (n=1) | Health Facility | Rural and Urban | Doctor | Not stated | Not stated | Not stated | Not stated | Not stated | Not stated | Not stated | Not stated | Not stated | Not stated | Not stated |
|  |  |  |  |  |  |  |  |  | Female Health Technician | Not stated | Not stated | Not stated | Not stated | Not stated | Not stated | Not stated | Not stated | Not stated | Not stated | Not stated |
|  |  |  |  |  |  |  |  |  | Lady Health Visitor | Not stated | Not stated | Not stated | Not stated | Not stated | Not stated | Not stated | Not stated | Not stated | Not stated | Not stated |
|  |  |  |  |  |  |  |  |  | Midwife | Not stated | Not stated | Not stated | Not stated | Not stated | Not stated | Not stated | Not stated | Not stated | Not stated | Not stated |
|  |  |  |  |  |  |  |  |  | Nurse | Not stated | Not stated | Not stated | Not stated | Not stated | Not stated | Not stated | Not stated | Not stated | Not stated | Not stated |
| [28] | Foster | 2006 | 2005 | Qualitative | Dominican Republic | Hospital (n=1) | Hospital | Urban | Auxiliary Nurse | Yes | Perform | Perform | Not stated | Perform | Not stated | Perform | Not stated | Not stated | Not stated | Secondary school + 1 year training |
|  |  |  |  |  |  |  |  |  | Registered Nurse | Yes | Not stated | Not stated | Not stated | Not stated | Not stated | Not stated | Not stated | Not stated | Not stated | Degree |
| [29] | Fujita | 2015 | 2012 | Cross-sectional | Zambia | Health Center (n=1) | Health Center | Not stated | Midwife | Not stated | Not stated | Not stated | Not stated | Not stated | Not stated | Not stated | Not stated | Not stated | Not stated | Not stated |
| [30] | Gebreegziabher | 2014 | 2014 | Cross-sectional | Ethiopia | Hospital (n=1) | Hospital | Urban | Midwife | Not stated | Not stated | Not stated | Not stated | Not stated | Not stated | Not stated | Some perform | Not stated | Not stated | Not stated |
|  |  |  |  |  |  |  |  |  | Nurse | Not stated | Not stated | Not stated | Not stated | Not stated | Not stated | Not stated | Some perform | Not stated | Not stated | Not stated |
|  |  |  |  |  |  |  |  |  | Obstetrician / Gynecologist | Not stated | Not stated | Not stated | Not stated | Not stated | Not stated | Not stated | Some perform | Not stated | Not stated | Not stated |
|  |  |  |  |  |  |  |  |  | Pediatrician | Not stated | Not stated | Not stated | Not stated | Not stated | Not stated | Not stated | Some perform | Not stated | Not stated | Not stated |
| [31] | Haile-Mariam | 2012 | 2008-2009 | Cross-sectional | Ethiopia | Health Facilities (n=741) | Health Facility | Rural and Urban | Midwife | Yes | Not stated | Not stated | Not stated | Not stated | Not stated | Not stated | Some perform | Not stated | Not stated | Not stated |
|  |  |  |  |  |  |  |  |  | Nurse | Yes | Not stated | Not stated | Not stated | Not stated | Not stated | Not stated | Some perform | Not stated | Not stated | Not stated |
| [32] | Hammah | 2013 | 2009-2010 | Mixed methods | Ghana | Health Facilities (n=2) in the city of Accra | Health Facility | Urban | Midwife | Yes | Not stated | Not stated | Not stated | Not stated | Not stated | Not stated | Not stated | Not stated | Not stated | Not stated |
| [33] | Harvey | 2004 | 2002 | Cross-sectional | Benin | National tertiary care facility (n=1), District level hospitals (at least n=2) and a mix of rural and urban facilities | Health Facility | Rural and Urban | Certified Midwife | Yes | Perform | Not stated | Perform | Perform | Not stated | Not stated | Some perform | Not stated | Not stated | Not stated |
|  |  |  |  |  |  |  |  |  | Doctor | Yes | Perform | Not stated | Perform | Perform | Not stated | Not stated | Some perform | Not stated | Not stated | Not stated |
|  |  |  |  |  | Ecuador | National tertiary care facility (n=1), District level hospitals (at least n=2) and a mix of rural and urban facilities | Health Facility | Rural and Urban | Certified Midwife | Yes | Perform | Not stated | Perform | Perform | Not stated | Not stated | Some perform | Not stated | Not stated | Not stated |
|  |  |  |  |  |  |  |  |  | Doctor | Yes | Perform | Not stated | Perform | Perform | Not stated | Not stated | Some perform | Not stated | Not stated | Not stated |
|  |  |  |  |  | Jamaica | National tertiary care facility (n=1), District level hospitals (at least n=2) and a mix of rural and urban facilities | Health Facility | Rural and Urban | Certified Midwife | Yes | Perform | Not stated | Perform | Perform | Not stated | Not stated | Some perform | Not stated | Not stated | Not stated |
|  |  |  |  |  |  |  |  |  | Certified Midwife | Yes | Perform | Not stated | Perform | Perform | Not stated | Not stated | Some perform | Not stated | Not stated | Not stated |
|  |  |  |  |  |  |  |  |  | Doctor | Yes | Perform | Not stated | Perform | Perform | Not stated | Not stated | Some perform | Not stated | Not stated | Not stated |
|  |  |  |  |  |  |  |  |  | Nurse | Not stated | Not stated | Not stated | Not stated | Not stated | Not stated | Not stated | Not stated | Not stated | Not stated | Not stated |
|  |  |  |  |  | Rwanda | National tertiary care facility (n=1), District level hospitals (at least n=2) and a mix of rural and urban facilities | Health Facility | Rural and Urban | Certified Midwife | Yes | Perform | Not stated | Perform | Perform | Not stated | Not stated | Some perform | Not stated | Not stated | Not stated |
|  |  |  |  |  |  |  |  |  | Doctor | Yes | Perform | Not stated | Perform | Perform | Not stated | Not stated | Some perform | Not stated | Not stated | Not stated |
|  |  |  |  |  |  |  |  |  | Nurse | Not stated | Not stated | Not stated | Not stated | Not stated | Not stated | Not stated | Not stated | Not stated | Not stated | Not stated |
| [34] | Huchon | 2014 | 2012 | Cross-sectional | Mali | Referral health centers (n=9) in Regions (n=2) | Health Center (Referral) | Rural | Doctor | Yes | Not stated | Not stated | Not stated | Not stated | Not stated | Not stated | Not stated | Not stated | Not stated | Diploma |
|  |  |  |  |  |  |  |  |  | Midwife | Yes | Not stated | Not stated | Not stated | Not stated | Not stated | Not stated | Not stated | Not stated | Not stated | Degree |
|  |  |  |  |  |  |  |  |  | Obstetric Nurse | Yes | Not stated | Not stated | Not stated | Not stated | Not stated | Not stated | Not stated | Not stated | Not stated | Primary school + 3 years training |
| [35] | Ith | 2013 | Not stated | Qualitative | Cambodia | Hospital (n=1), Referral Hospitals (n=2) and Health centers (n=2) in one Province | Health Facility | Not stated | Doctor | Yes | Not stated | Perform | Not stated | Perform | Perform | Perform | Not performed | Not stated | Not stated | Not stated |
|  |  |  |  |  |  |  |  |  | Nurse | Yes | Not stated | Perform | Not stated | Perform | Perform | Perform | Not performed | Not stated | Not stated | Secondary school + 1 year training |
|  |  |  |  |  |  |  |  |  | Primary Midwife | Yes | Not stated | Perform | Not stated | Perform | Perform | Perform | Not performed | Not stated | Not stated | Not stated |
|  |  |  |  |  |  |  |  |  | Secondary Midwife | Yes | Not stated | Perform | Not stated | Perform | Perform | Perform | Not performed | Not stated | Not stated | Not stated |
| [36] | Jones | 2015 | 2013 | Cross-sectional | Sierra Leone | National | Health Facility | Rural and Urban | Maternal and Child Health Aide | No | Not stated | Not stated | Not stated | Not stated | Not stated | Not stated | Not stated | Not stated | Not stated | Not stated |
| [37] | Jones | 2015 | Not stated | Qualitative | United Republic of Tanzania | Hospitals (n=2 government, n-1 large tertiary) in Dar es Salaam | Hospital | Urban | Nurse Midwife | Yes | Not stated | Not stated | Not stated | Not stated | Not stated | Not stated | Not stated | Not stated | Not stated | Degree |
| [38] | Kildea | 2012 | Not stated | Mixed methods | Mongolia | Districts (n=6) | Health Facility | Rural and Urban | Family Doctor | Not stated | Not stated | Not stated | Not stated | Not stated | Not stated | Not stated | Not stated | Not stated | Not stated | Not stated |
|  |  |  |  |  |  |  |  |  | Feldsher | Yes | Not stated | Not stated | Not stated | Not stated | Not stated | Not stated | Not stated | Not stated | Not stated | Degree |
|  |  |  |  |  |  |  |  |  | Midwife | Yes | Not stated | Not stated | Not stated | Not stated | Not stated | Not stated | Not stated | Not stated | Not stated | Degree |
|  |  |  |  |  |  |  |  |  | Neonatal Nurse | Not stated | Not stated | Not stated | Not stated | Not stated | Not stated | Not stated | Not stated | Not stated | Not stated | Not stated |
|  |  |  |  |  |  |  |  |  | Nurse | Not stated | Not stated | Not stated | Not stated | Not stated | Not stated | Not stated | Not stated | Not stated | Not stated | Not stated |
|  |  |  |  |  |  |  |  |  | Nurse | Not stated | Not stated | Not stated | Not stated | Not stated | Not stated | Not stated | Not stated | Not stated | Not stated | Not stated |
|  |  |  |  |  |  |  |  |  | Nurse Midwife | Yes | Not stated | Not stated | Not stated | Not stated | Not stated | Not stated | Not stated | Not stated | Not stated | Degree |
|  |  |  |  |  |  |  |  |  | Obstetrician / Gynecologist | Yes | Not stated | Not stated | Not stated | Not stated | Not stated | Not stated | Not stated | Not stated | Not stated | Secondary school + 3 years training |
| [39] | Kim | 2013 | 2009-2010 | Cross-sectional | Afghanistan | National | Health Facility | Rural and Urban | Doctor | Yes | Not stated | Not stated | Not stated | Not stated | Not stated | Not stated | Some perform | Not stated | Not stated | Not stated |
|  |  |  |  |  |  |  |  |  | Midwife | Yes | Not stated | Not stated | Not stated | Not stated | Not stated | Not stated | Some perform | Not stated | Not stated | Not stated |
| [40] | Knoble | 2010 | 2007 | Cross-sectional | Nepal | District (n=4) | Health Facility | Rural | Auxiliary Health Worker | Not stated | Not stated | Not stated | Not stated | Not stated | Not stated | Not stated | Not stated | Not stated | Not stated | Not stated |
|  |  |  |  |  |  |  |  |  | Health Assistant | Not stated | Not stated | Not stated | Not stated | Not stated | Not stated | Not stated | Not stated | Not stated | Not stated | Not stated |
|  |  |  |  |  |  |  |  |  | Senior Auxilary Health Worker | Not stated | Not stated | Not stated | Not stated | Not stated | Not stated | Not stated | Not stated | Not stated | Not stated | Not stated |
| [41] | Lobis | 2011 | 2009 | Mixed methods | Malawi | National | Hospital (District and Central) | Rural and Urban | Clinical Officer | Not stated | Perform | Perform | Perform | Perform | Perform | Perform | Perform | Perform | Perform | 1) 3 years training + 1 year internship or 2) 18 months upgrade from medical assistant + 1 year internship |
|  |  |  |  |  |  |  | Health Facility |  | Doctor | Not stated | Perform | Perform | Perform | Perform | Perform | Perform | Perform | Perform | Perform | Not stated |
|  |  |  |  |  |  |  | Health Facility |  | Enrolled Nurse Midwife | Not stated | Perform | Perform | Perform | Perform | Perform | Perform | Perform | Not stated | Not stated | Not stated |
|  |  |  |  |  |  |  | Hospital (District) and Health Center |  | Medical Assistant | Not stated | Perform | Perform | Perform | Some perform | Some perform | Some perform | Perform | Not stated | Not stated | Secondary school + 2 years training + 1 year internship |
|  |  |  |  |  |  |  | Health Facility |  | Nurse Midwife Technician | Not stated | Perform | Perform | Perform | Perform | Not performed | Perform | Perform | Not stated | Not stated | Not stated |
|  |  |  |  |  |  |  | Health Facility |  | Registered Nurse Midwife | Not stated | Perform | Perform | Perform | Perform | Perform | Perform | Perform | Not stated | Perform | Not stated |
|  |  |  |  |  | United Republic of Tanzania | National | Hospital (Regional and District) | Rural and Urban | Assistant Medical Officer | Not stated | Perform | Perform | Perform | Perform | Perform | Perform | Perform | Perform | Perform | Clinical officer designation + 2 years training |
|  |  |  |  |  |  |  | Health Center |  | Clinical Officer | Not stated | Perform | Perform | Perform | Perform | Perform | Not performed | Perform | Not stated | Perform | Two routes: 1) Secondary school + 3 years training or 2) Clinical Asssistant + 1 year training |
|  |  |  |  |  |  |  | Health Facility |  | Enrolled Nurse Midwife | Not stated | Perform | Perform | Perform | Perform | Perform | Perform | Perform | Not stated | Perform | Not stated |
|  |  |  |  |  |  |  | Health Facility |  | Medical Officer | Not stated | Perform | Perform | Perform | Perform | Perform | Perform | Perform | Perform | Perform | Not stated |
|  |  |  |  |  |  |  | Health Facility |  | Registered Nurse Midwife | Not stated | Perform | Perform | Perform | Perform | Perform | Perform | Perform | Not stated | Perform | Not stated |
| [42] | Makowiecka | 2008 | 2005 | Cross-sectional | Indonesia | District (n=2) | Hospital (Public) | Rural and Urban | Midwife | Yes | Not stated | Not stated | Not stated | Not stated | Not stated | Not stated | Not stated | Not stated | Not stated | Diploma |
|  |  |  |  |  |  |  | Health Center | Rural and Urban | Midwife | Yes | Not stated | Not stated | Not stated | Not stated | Not stated | Not stated | Not stated | Not stated | Not stated | Diploma |
|  |  |  |  |  |  |  | Hospital (Public) | Rural and Urban | Midwife | Yes | Not stated | Not stated | Not stated | Not stated | Not stated | Not stated | Not stated | Not stated | Not stated | Diploma |
|  |  |  |  |  |  |  | Community | Rural and Urban | Midwife | Yes | Not stated | Not stated | Not stated | Not stated | Not stated | Not stated | Not stated | Not stated | Not stated | Diploma |
|  |  |  |  |  |  |  | Health Facility (Public) | Rural and Urban | Nurse | Yes | Not stated | Not stated | Not stated | Not stated | Not stated | Not stated | Not stated | Not stated | Not stated | Diploma |
|  |  |  |  |  |  |  | Community | Rural and Urban | Village Midwife | Yes | Not stated | Not stated | Not stated | Not stated | Not stated | Not stated | Not stated | Not stated | Not stated | Diploma |
| [43] | Malhotra | 2014 | 2010 | Cross-sectional | India | District Hospital (n=1) and Community Health Centers (n=2) from Districts (n=2) | Hospital, Health Facility and Community | Rural and Urban | Auxiliary Nurse | Yes | Not stated | Not stated | Not stated | Not stated | Not stated | Perform | Perform | Not stated | Not stated | Not stated |
|  |  |  |  |  |  |  |  |  | Obstetrician / Pediatrician | Yes | Not stated | Not stated | Not stated | Not stated | Not stated | Perform | Perform | Not stated | Not stated | Not stated |
|  |  |  |  |  |  |  |  |  | Staff Nurse | Yes | Not stated | Not stated | Not stated | Not stated | Not stated | Perform | Perform | Not stated | Not stated | Not stated |
| [44] | Mamba | 2000 | Not stated | Cross-sectional | Swaziland | Antenatal clinics in public health units (n=6) in Regions (n=4) | Health Center or Health Clinic (Public) | Rural | Nurse Midwife | Not stated | Not stated | Not stated | Not stated | Not stated | Not stated | Not stated | Not stated | Not stated | Not stated | Not stated |
| [45] | Mangham-Jefferies | 2014 | 2008-2013 | Qualitative | Ethiopia | Rural kebeles (smallest admin structure of government) in SNNP region and n=2 zones of Oromia Region | Health Post | Rural | Health Extention Worker | Not stated | Not stated | Not stated | Not stated | Not stated | Not stated | Not stated | Not stated | Not stated | Not stated | Secondary school + 1 year training |
| [46] | Mansoor | 2013 | 2011-2012 | Cross-sectional | Afghanistan | Provinces (n=11) | Health Facility (Public) and Community | Rural | Midwife (CME) | Yes | Not stated | Not stated | Not stated | Perform | Perform | Perform | Not stated | Not stated | Not stated | Not stated |
|  |  |  |  |  |  |  | Hospital (Provincial and District) | Rural and Urban | Midwife (IHS) | Yes | Not stated | Not stated | Not stated | Perform | Perform | Perform | Not stated | Not stated | Not stated | Not stated |
| [47] | Mirkuzie | 2014 | 2013 | Cross-sectional | Ethiopia | Public Health Centers (n=10) in Addis Ababa | Health Center | Urban | Midwife | Not stated | Not stated | Not stated | Not stated | Not stated | Not stated | Not stated | Not stated | Not stated | Not stated | Not stated |
|  |  |  |  |  |  |  |  |  | Nurse | Not stated | Not stated | Not stated | Not stated | Not stated | Not stated | Not stated | Not stated | Not stated | Not stated | Not stated |
| [48] | Nsemo | 2013 | Not stated | Mixed methods | Nigeria | Hospitals (n=1) in a city | Hospital | Urban | Midwife | Not stated | Not stated | Not stated | Not stated | Not stated | Not stated | Not stated | Not stated | Not stated | Not stated | Degree |
|  |  |  |  |  |  |  |  |  | Nurse | Not stated | Not stated | Not stated | Not stated | Not stated | Not stated | Not stated | Not stated | Not stated | Not stated | Degree |
| [49] | Nyango | 2010 | Not stated | Cross-sectional | Nigeria | State (n=1) | Health Facility | Rural | Community Health Extension Worker | No | Not stated | Not stated | Not stated | Not stated | Not stated | Not stated | Not stated | Not stated | Not stated | Not stated |
|  |  |  |  |  |  |  |  |  | Doctor | Not stated | Not stated | Not stated | Not stated | Not stated | Not stated | Not stated | Not stated | Not stated | Not stated | Not stated |
|  |  |  |  |  |  |  |  |  | Nurse | Not stated | Not stated | Not stated | Not stated | Not stated | Not stated | Not stated | Not stated | Not stated | Not stated | Degree |
|  |  |  |  |  |  |  | Health Center (Public) | Rural | Nurse Midwife | Yes | Not stated | Perform | Not stated | Some perform | Not stated | Not stated | Not stated | Not stated | Not stated | Not stated |
| [50] | Ogunlesi | 2008 | 2006 | Cross-sectional | Nigeria | Hospitals (n=4) in States (n=4) in the Southwestern Nigeria | Hospital | Rural and Urban | Nurse | Not stated | Not stated | Not stated | Not stated | Not stated | Not stated | Not stated | Not stated | Not stated | Not stated | Not stated |
| [51] | Ojofeitimi | 2009 | Not stated | Cross-sectional | Nigeria | Health institutions (n=2) in one State | Health Facility | Not stated | Community Health Officer | Not stated | Not stated | Not stated | Not stated | Not stated | Not stated | Not stated | Not stated | Not stated | Not stated | Secondary school |
|  |  |  |  |  |  |  |  |  | Medical Student | Not stated | Not stated | Not stated | Not stated | Not stated | Not stated | Not stated | Not stated | Not stated | Not stated | Post-secondary in service training |
|  |  |  |  |  |  |  |  |  | Nursing Officer | Not stated | Not stated | Not stated | Not stated | Not stated | Not stated | Not stated | Not stated | Not stated | Not stated | Post-secondary |
| [52] | Oladapo | 2006 | 2004-2005 | Cross-sectional | Nigeria | State (n=1) peripheral maternity units (n=66) | Health Center | Rural and Urban | Community Health Officer | Not stated | Not stated | Not stated | Not stated | Not stated | Not stated | Not stated | Not stated | Not stated | Not stated | Not stated |
|  |  |  |  |  |  |  |  |  | Health Attendant | Not stated | Not stated | Not stated | Not stated | Not stated | Not stated | Not stated | Not stated | Not stated | Not stated | Not stated |
|  |  |  |  |  |  |  |  |  | Junior Community Heatlh Extension Worker | Not stated | Not stated | Not stated | Not stated | Not stated | Not stated | Not stated | Not stated | Not stated | Not stated | Not stated |
|  |  |  |  |  |  |  |  |  | Matron | Not stated | Not stated | Not stated | Not stated | Not stated | Not stated | Not stated | Not stated | Not stated | Not stated | Not stated |
|  |  |  |  |  |  |  |  |  | Medical Officer of Health | Not stated | Not stated | Not stated | Not stated | Not stated | Not stated | Not stated | Not stated | Not stated | Not stated | Not stated |
|  |  |  |  |  |  |  |  |  | Senior Community Health Extention Worker | Not stated | Not stated | Not stated | Not stated | Not stated | Not stated | Not stated | Not stated | Not stated | Not stated | Not stated |
|  |  |  |  |  |  |  |  |  | Senior Community Health Officer | Not stated | Not stated | Not stated | Not stated | Not stated | Not stated | Not stated | Not stated | Not stated | Not stated | Not stated |
|  |  |  |  |  |  |  |  |  | Senior Nursing Sister | Not stated | Not stated | Not stated | Not stated | Not stated | Not stated | Not stated | Not stated | Not stated | Not stated | Not stated |
|  |  |  |  |  |  |  |  |  | Staff Nurse | Not stated | Not stated | Not stated | Not stated | Not stated | Not stated | Not stated | Not stated | Not stated | Not stated | Not stated |
| [53] | Oladapo | 2009 | 1999-2000 | Cross-sectional | Nigeria | Health Faculties (n=7) in one Southwest Region | Hospital and Health Facility (Public, Tertiary Care) | Rural and Urban | Doctor (Intern) | Not stated | Some perform | Not stated | Not stated | Some perform | Not stated | Not stated | Not stated | Not stated | Some perform | Not stated |
|  |  |  |  |  |  |  |  |  | Matron | Not stated | Some perform | Not stated | Not stated | Some perform | Not stated | Not stated | Not stated | Not stated | Some perform | Not stated |
|  |  |  |  |  |  |  |  |  | Nurse Midwife | Not stated | Some perform | Not stated | Not stated | Some perform | Not stated | Not stated | Not stated | Not stated | Some perform | Not stated |
|  |  |  |  |  |  |  |  |  | Obstetrician / Gynecologist | Not stated | Some perform | Not stated | Not stated | Some perform | Not stated | Not stated | Not stated | Not stated | Some perform | Not stated |
|  |  |  |  |  |  |  |  |  | Resident Doctor | Not stated | Some perform | Not stated | Not stated | Some perform | Not stated | Not stated | Not stated | Not stated | Some perform | Not stated |
| [54] | Opiah | 2012 | Not stated | Cross-sectional | Nigeria | Health facilities (n=2) | Hospital | Urban | Midwife | Yes | Not stated | Not stated | Not stated | Not stated | Not stated | Not stated | Not stated | Not stated | Not stated | Not stated |
| [55] | Partamin | 2012 | 2009-2010 | Cross-sectional | Afghanistan | CEMONC health facilities (n=78) | Health Facility | Rural and Urban | Doctor | Yes | Perform | Not stated | Not stated | Not stated | Not stated | Perform | Perform | Not stated | Not stated | Not stated |
|  |  |  |  |  |  |  |  |  | Midwife | Yes | Perform | Not stated | Not stated | Not stated | Not stated | Perform | Perform | Not stated | Not stated | Not stated |
| [56] | Parveen | 2011 | 2007-2008 | Mixed methods | Bangladesh | Hospitals (n=6) from Districts (n=2) | Hospital | Not stated | Assistant Midwife | Yes | Not stated | Not stated | Not stated | Not stated | Not stated | Not stated | Not stated | Not stated | Not stated | Not stated |
|  |  |  |  |  |  |  |  |  | Community Skilled Birth Attendant | Yes | Not stated | Not stated | Not stated | Not stated | Not stated | Not stated | Not stated | Not stated | Not stated | Degree |
|  |  |  |  |  |  |  |  |  | Family Welfare Visitor | Yes | Not stated | Not stated | Not stated | Not stated | Not stated | Not stated | Not stated | Not stated | Not stated | Not stated |
|  |  |  |  |  |  |  |  |  | Medical Assistant | Yes | Not stated | Not stated | Not stated | Not stated | Not stated | Not stated | Not stated | Not stated | Not stated | Not stated |
|  |  |  |  |  |  |  |  |  | Nurse | Yes | Not stated | Not stated | Not stated | Not stated | Not stated | Not stated | Not stated | Not stated | Not stated | Not stated |
| [57] | Premadasa | 2008 | 2005-2006 | Cross-sectional | Kuwait | National | Health Facility | Rural and Urban | Student Medical Officer | Not stated | Not stated | Not stated | Not stated | Not stated | Not stated | Not stated | Not stated | Not stated | Not stated | Not stated |
| [58] | Prytherch | 2012 | 2010 | Cross-sectional | United Republic of Tanzania | Health Facilities (n=10 public, n=2 faith based) in Districts (n=2). | Health Facility | Rural | Assistant Health Officer | Not stated | Not stated | Not stated | Not stated | Not stated | Not stated | Not stated | Not stated | Not stated | Not stated | Clinical officer designation + 3 years training |
|  |  |  |  |  |  |  |  |  | Assistant Medical Officer | Not stated | Not stated | Not stated | Not stated | Not stated | Not stated | Not stated | Not stated | Not stated | Not stated | Secondary school + 2 years training |
|  |  |  |  |  |  |  |  |  | Clinical Officer | Not stated | Not stated | Not stated | Not stated | Not stated | Not stated | Not stated | Not stated | Not stated | Not stated | Secondary school + 3 years training |
|  |  |  |  |  |  |  |  |  | Doctor (Specialist) | Not stated | Not stated | Not stated | Not stated | Not stated | Not stated | Not stated | Not stated | Not stated | Not stated | Degree + residency training |
|  |  |  |  |  |  |  |  |  | Enrolled Nurse | Not stated | Not stated | Not stated | Not stated | Not stated | Not stated | Not stated | Not stated | Not stated | Not stated | In-service training as nurse midwife |
|  |  |  |  |  |  |  |  |  | Enrolled Nurse Midwife | Not stated | Not stated | Not stated | Not stated | Not stated | Not stated | Not stated | Not stated | Not stated | Not stated | Diploma (4 years) or Degree (3 years) |
|  |  |  |  |  |  |  |  |  | Maternal and Child Health Aide | Not stated | Not stated | Not stated | Not stated | Not stated | Not stated | Not stated | Not stated | Not stated | Not stated | Pre-service training |
|  |  |  |  |  |  |  |  |  | Nurse Midwife | Not stated | Not stated | Not stated | Not stated | Not stated | Not stated | Not stated | Not stated | Not stated | Not stated | Secondary school + 3 years training |
|  |  |  |  |  |  |  |  |  | Nursing Attendant | Not stated | Not stated | Not stated | Not stated | Not stated | Not stated | Not stated | Not stated | Not stated | Not stated | Secondary + 2 years training |
|  |  |  |  |  |  |  |  |  | Public Health Nurse | Not stated | Not stated | Not stated | Not stated | Not stated | Not stated | Not stated | Not stated | Not stated | Not stated | Secondary school + 2 years training |
|  |  |  |  |  |  |  |  |  | Registered Nurse | Not stated | Not stated | Not stated | Not stated | Not stated | Not stated | Not stated | Not stated | Not stated | Not stated | Secondary school + 3 years training |
| [59] | Sarfraz | 2014 | 2011 | Qualitative | Pakistan | Tehsils (n=6, second-lowest tier of local government, 5 rural, 1 urban) from one district | Community | Rural and Urban | Community Midwife | Yes | Not stated | Not stated | Not stated | Not stated | Not stated | Perform | Not stated | Not stated | Not stated | Community based training (12 months) |
|  |  |  |  |  |  |  |  |  | Lady Health Visitor | Yes | Not stated | Not stated | Not stated | Not stated | Not stated | Not stated | Not stated | Not stated | Not stated | Not stated |
|  |  |  |  |  |  |  |  |  | Lady Health Worker | Yes | Not stated | Not stated | Not stated | Not stated | Not stated | Not stated | Not stated | Not stated | Not stated | Diploma in public health nursing |
| [60] | Saswata | 2005 | 2003 | Cross-sectional | Uganda | National | Health Facility | Rural and Urban | Clinical Officer | Not stated | Not stated | Not stated | Not stated | Some perform | Not stated | Not stated | Not stated | Not stated | Not stated | Specialist training (3 years) |
| [61] | Schack | 2014 | 2011 | Qualitative | Ghana | District hospitals (n=2) | Hospital | Urban | Midwife | Not stated | Not stated | Not stated | Not stated | Not stated | Not stated | Not stated | Not stated | Not stated | Not stated | Not stated |
| [62] | Shaban | 2012 | 2009 | Mixed methods | Jordan | National | Health Facility | Not stated | Midwife | Yes | Not stated | Not stated | Not stated | Not stated | Not stated | Not stated | Not stated | Not stated | Not stated | Degree |
| [63] | Sharma | 2013 | 2008-2009 | Mixed methods | India | Maternity sections of public health facilities in a province | Community | Rural | Auxiliary Nurse Midwife | Yes | Not stated | Not stated | Not stated | Not stated | Not stated | Not stated | Not stated | Not stated | Not stated | Secondary school + 1.5 years training |
|  |  |  |  |  |  |  |  |  | Graduate Nurse | Yes | Not stated | Not stated | Not stated | Not stated | Not stated | Not stated | Not stated | Not stated | Not stated | Diploma in midwifery |
|  |  |  |  |  |  |  |  |  | Staff Nurse | Yes | Not stated | Not stated | Not stated | Perform | Not stated | Not stated | Not stated | Not stated | Not stated | Degree + additional 11 months to qualify as specialized midwives |
| [64] | Shimoda | 2015 | Not stated | Qualitative | United Republic of Tanzania | Hospital (n=1) and health center (n=1) in Dar es Salaam | Health Facility | Urban | Midwife | Not stated | Not stated | Not stated | Not stated | Not stated | Not stated | Not stated | Not stated | Not stated | Not stated | Not stated |
| [65] | Syamala | 2004 | Not stated | Mixed methods | India | Districts (n=11) in one State | Community | Rural | Multipurpose Health Worker (Female) - MPHW (F)/ANM | Not stated | Not stated | Not stated | Not stated | Not stated | Not stated | Not stated | Not stated | Not stated | Not stated | Assistant Nurse Midwifery (basic) training |
| [66] | Traore | 2014 | 2011-2012 | Cross-sectional | Mali | Districts (n=2) | Health Facility | Rural and Urban | Doctor | Yes | Not stated | Not stated | Not stated | Not stated | Not stated | Not stated | Not stated | Not stated | Not stated | Degree + residency training |
|  |  |  |  |  |  |  |  |  | Health Technician | Not stated | Not stated | Not stated | Not stated | Not stated | Not stated | Not stated | Not stated | Not stated | Not stated | Secondary school + 3 years training |
|  |  |  |  |  |  |  |  |  | Matron | Not stated | Not stated | Not stated | Not stated | Not stated | Not stated | Not stated | Not stated | Not stated | Not stated | Primary school + 3 years training |
|  |  |  |  |  |  |  |  |  | Midwife | Yes | Not stated | Not stated | Not stated | Not stated | Not stated | Not stated | Not stated | Not stated | Not stated | Primary school + 3 years training |
|  |  |  |  |  |  |  |  |  | Obstetric Nurse | Yes | Not stated | Not stated | Not stated | Not stated | Not stated | Not stated | Not stated | Not stated | Not stated | Secondary school + 3 years training |
|  |  |  |  |  |  |  |  |  | Senior Health Technician | Yes | Not stated | Not stated | Not stated | Not stated | Not stated | Not stated | Not stated | Not stated | Not stated | No formal education |
| [67] | Ueno | 2015 | 2012 | Cross-sectional | United Republic of Tanzania | District (n=1) | Health Facility | Urban | Assistant Clinical Officer | No | Perform | Perform | Not performed | Not performed | Not performed | Not performed | Not performed | Not performed | Not performed | Secondary school + 2 years training |
|  |  |  |  |  |  |  |  |  | Assistant Medical Officer | Yes | Perform | Perform | Some perform | Some perform | Some perform | Some perform | Some perform | Some perform | Some perform | Not stated |
|  |  |  |  |  |  |  |  |  | Clinical Officer | Yes | Some perform | Perform | Some perform | Some perform | Some perform | Not performed | Some perform | Not performed | Some perform | Secondary school + 3 years training |
|  |  |  |  |  |  |  |  |  | Doctor | Yes | Some perform | Some perform | Some perform | Some perform | Perform | Some perform | Some perform | Perform | Some perform | Not stated |
|  |  |  |  |  |  |  |  |  | Enrolled Nurse | Yes | Not stated | Not stated | Not stated | Not stated | Not stated | Not stated | Not stated | Not stated | Not stated | Not stated |
|  |  |  |  |  |  |  |  |  | Enrolled Nurse Midwife | Yes | Some perform | Perform | Some perform | Some perform | Some perform | Some perform | Some perform | Not performed | Some perform | Nurse/Midwifery school + 2 years additional training |
|  |  |  |  |  |  |  |  |  | Maternal and Child Health Aide | Yes | Perform | Perform | Not performed | Not performed | Not performed | Not performed | Perform | Not performed | Not performed | Professional Nursing or Midwifery education (4-5 years) |
|  |  |  |  |  |  |  |  |  | Medical Assistant | No | Some perform | Some perform | Some perform | Not performed | Not performed | Not performed | Some perform | Not performed | Some perform | Midwifery course (18 months) |
|  |  |  |  |  |  |  |  |  | Obstetrician / Gynecologist | Yes | Perform | Perform | Perform | Perform | Some perform | Perform | Perform | Perform | Perform | Not stated |
|  |  |  |  |  |  |  |  |  | Public Health Nurse | Yes | Perform | Perform | Not performed | Not performed | Not performed | Not performed | Not performed | Not performed | Not performed | Not stated |
|  |  |  |  |  |  |  |  |  | Registered Nurse Midwife | Yes | Some perform | Some perform | Some perform | Some perform | Some perform | Some perform | Some perform | Not performed | Some perform | Diploma in nursing + midwifery |
| [68] | Utz | 2013 | 2011-2012 | Cross-sectional | Bangladesh | Key informants from each country | Health Facility | Rural and Urban | Assistant Nurse | No | Not stated | Not stated | Not stated | Not stated | Not stated | Not stated | Not stated | Not stated | Not stated | Not stated |
|  |  |  |  |  |  |  |  |  | Community Skilled Birth Attendant | Yes | Perform | Perform | Perform | Not performed | Not performed | Not performed | Perform | Not performed | Not performed | Degree |
|  |  |  |  |  |  |  |  |  | Family Welfare Visitor | Yes | Perform | Perform | Perform | Not performed | Not performed | Not performed | Perform | Not performed | Not performed | Degree |
|  |  |  |  |  |  |  |  |  | Medical Assistant | No | Not stated | Not stated | Not stated | Not stated | Not stated | Not stated | Not stated | Not stated | Not stated | Degree + 1 year training in obstetrics |
|  |  |  |  |  |  |  |  |  | Medical Officer | No | Perform | Perform | Perform | Not performed | Not performed | Not performed | Perform | Not performed | Perform | No formal education |
|  |  |  |  |  |  |  |  |  | Medical Officer - Obstetrics trained | Yes | Perform | Perform | Perform | Perform | Perform | Perform | Perform | Perform | Perform | Not stated |
|  |  |  |  |  |  |  |  |  | Midwife | No | Not stated | Not stated | Not stated | Not stated | Not stated | Not stated | Not stated | Not stated | Not stated | Not stated |
|  |  |  |  |  |  |  |  |  | Obstetrician / Gynecologist | Yes | Perform | Perform | Perform | Perform | Perform | Perform | Perform | Perform | Perform | Not stated |
|  |  |  |  |  |  |  |  |  | Senior Staff Nurse | Yes | Perform | Perform | Perform | Not performed | Not performed | Not performed | Perform | Not performed | Not performed | Not stated |
|  |  |  |  |  |  |  |  |  | Staff Nurse | Yes | Perform | Perform | Perform | Not performed | Not performed | Not performed | Perform | Not performed | Not performed | Not stated |
|  |  |  |  |  |  |  |  |  | Sub-Assistant Community Medical Officer | No | Not stated | Not stated | Not stated | Not stated | Not stated | Not stated | Not stated | Not stated | Not stated | Not stated |
|  |  |  |  |  | India | Key informants from each country | Health Facility | Rural and Urban | Auxiliary Nurse Midwife | Yes | Perform | Perform | Perform | Not performed | Not performed | Not performed | Perform | Not performed | Not performed | Not stated |
|  |  |  |  |  |  |  |  |  | Ayurvedic Doctor | Yes | Perform | Perform | Perform | Not performed | Not performed | Not performed | Perform | Not performed | Not performed | Degree |
|  |  |  |  |  |  |  |  |  | Lady Health Visitor | Yes | Perform | Perform | Perform | Not performed | Not performed | Not performed | Perform | Not performed | Not performed | Degree + 16 weeks EMOC or 18 weeks for anesthesia training |
|  |  |  |  |  |  |  |  |  | Medical Officer | Yes | Perform | Perform | Perform | Perform | Perform | Perform | Perform | Perform | Perform | Degree + residency training |
|  |  |  |  |  |  |  |  |  | Medical Officer - Obstetrics trained | Yes | Perform | Perform | Perform | Perform | Perform | Perform | Perform | Perform | Perform | No formal education |
|  |  |  |  |  |  |  |  |  | Midwife | No | Not stated | Not stated | Not stated | Not stated | Not stated | Not stated | Not stated | Not stated | Not stated | Not stated |
|  |  |  |  |  |  |  |  |  | Obstetrician / Gynecologist | Yes | Perform | Perform | Perform | Perform | Perform | Perform | Perform | Perform | Perform | Not stated |
|  |  |  |  |  |  |  |  |  | Senior Staff Nurse | Yes | Perform | Perform | Perform | Not performed | Not performed | Not performed | Perform | Not performed | Perform | Assistant Nurse Midwife training + 6 monhs addition training |
|  |  |  |  |  |  |  |  |  | Staff Nurse | Yes | Perform | Perform | Perform | Not performed | Not performed | Not performed | Perform | Not performed | Perform | Not stated |
|  |  |  |  |  | Nepal | Key informants from each country | Health Facility | Rural and Urban | Auxiliary Nurse Midwife | Yes | Perform | Perform | Perform | Not performed | Not performed | Not performed | Perform | Not performed | Perform | Not stated |
|  |  |  |  |  |  |  |  |  | Generalist Physician | Yes | Perform | Perform | Perform | Perform | Perform | Perform | Perform | Perform | Perform | Degree |
|  |  |  |  |  |  |  |  |  | Maternal and Child Health Worker | No | Not stated | Not stated | Not stated | Not stated | Not stated | Not stated | Not stated | Not stated | Not stated | Degree + 6 months training in obstetrics |
|  |  |  |  |  |  |  |  |  | Medical Assistant | Yes | Perform | Perform | Perform | Not performed | Perform | Perform | Perform | Not performed | Perform | Degree + residency training |
|  |  |  |  |  |  |  |  |  | Medical Officer | No | Not stated | Not stated | Not stated | Not stated | Not stated | Not stated | Not stated | Not stated | Not stated | Degree + 3 year training in obstetrics |
|  |  |  |  |  |  |  |  |  | Medical Officer - Obstetrics trained | Yes | Perform | Perform | Perform | Perform | Perform | Perform | Perform | Perform | Perform | Not stated |
|  |  |  |  |  |  |  |  |  | Midwife | No | Not stated | Not stated | Not stated | Not stated | Not stated | Not stated | Not stated | Not stated | Not stated | No formal education |
|  |  |  |  |  |  |  |  |  | Obstetrician / Gynecologist | Yes | Perform | Perform | Perform | Perform | Perform | Perform | Perform | Perform | Perform | Not stated |
|  |  |  |  |  |  |  |  |  | Senior Staff Nurse | Yes | Perform | Perform | Perform | Not performed | Perform | Perform | Perform | Not performed | Perform | Not stated |
|  |  |  |  |  |  |  |  |  | Staff Nurse | Yes | Perform | Perform | Perform | Not performed | Not stated | Not performed | Perform | Not performed | Perform | Not stated |
|  |  |  |  |  | Pakistan | Key informants from each country | Health Facility | Rural and Urban | Community Skilled Birth Attendant | Yes | Perform | Perform | Perform | Not performed | Not performed | Not performed | Perform | Not performed | Not performed | Degree |
|  |  |  |  |  |  |  |  |  | Family Health Worker | No | Not stated | Not stated | Not stated | Not stated | Not stated | Not stated | Not stated | Not stated | Not stated | Degree + 1 year training in obstetrics |
|  |  |  |  |  |  |  |  |  | Lady Health Visitor | Yes | Perform | Perform | Perform | Not performed | Not performed | Not performed | Perform | Not performed | Perform | Degree + residency training |
|  |  |  |  |  |  |  |  |  | Medical Assistant | Yes | Perform | Perform | Perform | Perform | Perform | Perform | Perform | Perform | Perform | Not stated |
|  |  |  |  |  |  |  |  |  | Medical Officer | Yes | Perform | Perform | Perform | Perform | Perform | Perform | Perform | Perform | Perform | Degree + 18 months training |
|  |  |  |  |  |  |  |  |  | Medical Officer - Obstetrics trained | Yes | Perform | Perform | Perform | Perform | Perform | Perform | Perform | Perform | Perform | Not stated |
|  |  |  |  |  |  |  |  |  | Midwife | Yes | Perform | Perform | Perform | Not performed | Not performed | Not performed | Perform | Not performed | Perform | Not stated |
|  |  |  |  |  |  |  |  |  | Obstetrician / Gynecologist | Yes | Perform | Perform | Perform | Perform | Perform | Perform | Perform | Perform | Perform | Not stated |
|  |  |  |  |  |  |  |  |  | Senior Staff Nurse | Yes | Perform | Perform | Perform | Not performed | Not performed | Not performed | Perform | Not performed | Perform | Not stated |
|  |  |  |  |  |  |  |  |  | Staff Nurse | Yes | Perform | Perform | Perform | Not performed | Not performed | Not performed | Not performed | Not performed | Perform | Not stated |
| [69] | Yisma | 2013 | 2012 | Cross-sectional | Ethiopia | Addis Ababa | Health Facility | Urban | Doctor | Yes | Not stated | Not stated | Not stated | Not stated | Not stated | Not stated | Not stated | Not stated | Not stated | Not stated |
|  |  |  |  |  |  |  |  |  | Midwife | Yes | Not stated | Not stated | Not stated | Not stated | Not stated | Not stated | Not stated | Not stated | Not stated | Not stated |
|  |  |  |  |  |  |  |  |  | Nurse | Yes | Not stated | Not stated | Not stated | Not stated | Not stated | Not stated | Not stated | Not stated | Not stated | Not stated |
|  |  |  |  |  |  |  |  |  | Public Health Officer | Yes | Not stated | Not stated | Not stated | Not stated | Not stated | Not stated | Not stated | Not stated | Not stated | Not stated |
|  | Zainullah | 2014 | 2008-2010 | Mixed methods | Afghanistan | National | Hospital | Urban | Midwife | Yes | Not stated | Not stated | Not stated | Not stated | Not stated | Not stated | Not stated | Not stated | Not stated | Secondary school + 2 years training |
| [70] |  |  |  |  |  |  | Community | Rural | Midwife | Yes | Not stated | Not stated | Not stated | Not stated | Not stated | Not stated | Not stated | Not stated | Not stated | Secondary school + 2 years training |

**Reference ID**

1. Adegoke A, Utz B, Msuya SE, van den Broek N. Skilled Birth Attendants: who is who? A descriptive study of definitions and roles from nine Sub Saharan African countries. PloS one. 2012;7(7):e40220.

2. Adegoke AAC, Malcolm;Ogundeji, Martins O.;Lawoyin, Taiwo;Thomson, Ann M. Place of birth or place of death: An evaluation of 1139 maternal deaths in Nigeria. Midwifery. 2013;29(11):e115-21 1p.

3. Anderson FWJO, Samuel A.;Boothman, Erika L.;Opare-Ado, Henry. The Public Health Impact of Training Physicians to Become Obstetricians and Gynecologists in Ghana. American Journal of Public Health. 2014;104(S1):S159-65 1p.

4. Ariff SS, Sajid B.;Sadiq, Kamran;Feroze, Asher B.;Khan, Shuaib;Jafarey, Sadiqua N.;Ali, Nabeela;Bhutta, Zulfiqar A. Evaluation of health workforce competence in maternal and neonatal issues in public health sector of Pakistan: an Assessment of their training needs. BMC Health Services Research. 2010;10:319- 1p.

5. Ayiasi RM, Criel B, Orach CG, Nabiwemba E, Kolsteren P. Primary healthcare worker knowledge related to prenatal and immediate newborn care: a cross sectional study in Masindi, Uganda. BMC Health Services Research. 2014;14(1):65- 1p.

6. Bharati SI, Hildingsson;Eva, Johansson;Malvarappu, Prakasamma;K. V. Ramani;Kyllike, Christensson. Do the pre-service education programmes for midwives in India prepare confident ‘registered midwives’? A survey from India. Global Health Action. 2015;8:1-9.

7. Bhuiyan AB, Mukherjee S, Acharya S, Haider SJ, Begum F. Evaluation of a Skilled Birth Attendant pilot training program in Bangladesh. International journal of gynaecology and obstetrics: the official organ of the International Federation of Gynaecology and Obstetrics. 2005;90(1):56-60.

8. Bogren MUvT, Edwin;Berg, Marie. Where midwives are not yet recognised: A feasibility study of professional midwives in Nepal. Midwifery. 2013;29(10):1103-9 7p.

9. Bradley S, McAuliffe E. Mid-level providers in emergency obstetric and newborn health care: factors affecting their performance and retention within the Malawian health system. Human resources for health. 2009;7:14- 1p.

10. Carlo WA, Wright LL, Chomba E, McClure EM, Carlo ME, Bann CM, et al. Educational impact of the neonatal resuscitation program in low-risk delivery centers in a developing country. Journal of Pediatrics. 2009;154(4):504-8.e5 1p.

11. Carlough MM, M. Skilled birth attendance: what does it mean and how can it be measured? A clinical skills assessment of maternal and child health workers in Nepal. International journal of gynaecology and obstetrics: the official organ of the International Federation of Gynaecology and Obstetrics. 2005;89(2):200-8.

12. Cheung NF, Zhang L, Mander R, Xu X, Wang X. Proposed continuing professional education programme for midwives in China: New mothers' and midwives' views. Nurse Education Today. 2011;31(5):434-8 5p.

13. Danda G, Dube K, Dube P, Mudokwenyu-Rawdon C, Bedwell C. An observational study of midwives’ practices to prevent peripartum sepsis in Zimbabwe. African Journal of Midwifery & Women's Health. 2015;9(1):17-21 5p.

14. Danishevski K, McKee M, Balabanova D. Variations in obstetric practice in Russia: a story of professional autonomy, isolation and limited evidence. International Journal of Health Planning & Management. 2009;24(2):161-71 11p.

15. Dawson AK, M.;Geita, L.;Mola, G.;Brodie, P. M.;Rumsey, M.;Copeland, F.;Neill, A.;Homer, C. S. Midwifery capacity building in Papua New Guinea: Key achievements and ways forward. Women Birth. 2015.

16. Del Hierro GSR, R.;Verhoeven, V.;Van Royen, P.;Hendrickx, K. Are recent graduates enough prepared to perform obstetric skills in their rural and compulsory year? A study from Ecuador. BMJ open. 2014;4(7).

17. Demaria LM, Campero L, Vidler M, Walker D. Non-physician providers of obstetric care in Mexico: Perspectives of physicians, obstetric nurses and professional midwives. 2012;10:6.

18. Dgedge MM, Angel;Necochea, Edgar;Bossemeyer, Debora;Rajabo, Maharifa;Fullerton, Judith. Assessment of the nursing skill mix in Mozambique using a task analysis methodology. Human resources for health. 2014;12(1):5- 1p.

19. Dickerson AEF, Jennifer W.;Andes, Karen L. A profile of midwifery in Paraguay. Midwifery. 2014;30(10):1048-55 8p.

20. Dogba MF, Pierre;Berthe-Cisse, Safoura. Qualification of Staff, Organization of Services, and Management of Pregnant Women in Rural Settings: The Case of Diema and Kayes Districts (Mali). ISRN Obstetrics & Gynecology. 2012:8p-p 1p.

21. Erlandsson KS, Jamuna Tamrakar;Sapkota, Sabitri. Safety before comfort: a focused enquiry of Nepal skilled birth attendants' concepts of respectful maternity care. Evidence Based Midwifery. 2014;12(2):59-64 6p.

22. Evans CLJ, P.;Bazant, E.;Bhatnagar, N.;Zgambo, J.;Khamis, A. R. Competency-based training "Helping Mothers Survive: Bleeding after Birth" for providers from central and remote facilities in three countries. International journal of gynaecology and obstetrics: the official organ of the International Federation of Gynaecology and Obstetrics. 2014;126(3):286-90.

23. Ezeonwu MC. Maternal Birth Outcomes: Processes and Challenges in Anambra State, Nigeria. Health Care for Women International. 2011;32(6):492-514 23p.

24. Fawole AOK, K. I.;Adekanle, D. A. Knowledge and utilization of the partograph among obstetric care givers in south west Nigeria. African Journal of Reproductive Health. 2008;12(1):22-9 8p.

25. Ferdous JM, D.;Huda, F.;Quaiyum, M. A.;Anwar, I.;Koblinsky, M. Are nurses in obstetric wards in Bangladesh competent skilled birth attendants? : Dhaka, Bangladesh, International Centre for Diarrhoeal Disease Research, Bangladesh [ICDDR,B], Centre for Reproductive Health, 2011 Dec.; 2011.

26. Fichardt AEV, M. J. Assessment of learning needs and the development of an educational programme for registered nurses in advanced midwifery and neonatology. Curationis. 2000;23(4):107-16 10p.

27. Fikree FF, Mir AM, Haq IU. She may reach a facility but will still die! An analysis of quality of public sector maternal health services, District Multan, Pakistan. J Pak Med Assoc. 2006;56(4):156-63.

28. Foster JR, Y.;Heath, A. Decision making by auxiliary nurses to assess postpartum bleeding in a Dominican Republic maternity ward. JOGNN: Journal of Obstetric, Gynecologic & Neonatal Nursing. 2006;35(6):728-34 7p.

29. Fujita W, Mukumbuta L, Chavuma R, Ohashi K. Quality of partogram monitoring at a primary health centre in Zambia. Midwifery. 2015;31(1):191-6 6p.

30. Gebreegziabher E, Aregawi A, Getinet H. Knowledge and skills of neonatal resuscitation of health professionals at a university teaching hospital of Northwest Ethiopia. World Journal of Emergency Medicine. 2014;5(3):229-33.

31. Haile-Mariam AT, N.;Otterness, C.;Bailey, P. E. Assessing the health system's capacity to conduct neonatal resuscitation in Ethiopia. Ethiop Med J. 2012;50(1):43-55.

32. Hammah J, Donkor ES. Assessment of Practising Midwives on the Management of the Third Stage of Labour. African Journal of Midwifery and Women's Health. 2013;7(2):59-64.

33. Harvey SAA, P.;Bucagu, M.;Djibrina, S.;Edson, W. N.;Gbangbade, S.;McCaw-Binns, A.;Burkhalter, B. R. Skilled birth attendant competence: an initial assessment in four countries, and implications for the Safe Motherhood movement. International journal of gynaecology and obstetrics: the official organ of the International Federation of Gynaecology and Obstetrics. 2004;87(2):203-10.

34. Huchon C, Arsenault C, Tourigny C, Coulibaly A, Traore M, Dumont A, et al. Obstetric competence among referral healthcare providers in Mali. International journal of gynaecology and obstetrics: the official organ of the International Federation of Gynaecology and Obstetrics. 2014;126(1):56-9.

35. Ith PD, Angela;Homer, Caroline S. E.;Whelan, Anna Klinken. Practices of skilled birth attendants during labour, birth and the immediate postpartum period in Cambodia. Midwifery. 2013;29(4):300-7 8p.

36. Jones SA, C. A.;Gopalakrishnan, S.;Sam, B.;Bull, F.;Labicane, R. R.;Dabo, F.;den Broek, N. V. Building capacity for skilled birth attendance: An evaluation of the Maternal and Child Health Aides training programme in Sierra Leone. Midwifery. 2015.

37. Jones BM, R.;Butt, J.;Hauck, Y. Tanzanian midwives' perception of their professional role and implications for continuing professional development education. 2015.

38. Kildea S, Larsson M, Govind S. A review of midwifery in Mongolia utilising the ‘Strengthening Midwifery Toolkit’. Women & Birth. 2012;25(4):166-73 8p.

39. Kim YMvR, J.;Stekelenburg, J.;Ansari, N.;Kols, A.;Tappis, H.;Currie, S.;Zainullah, P.;Bailey, P.;Semba, R.;Sun, K. Assessing the capacity for newborn resuscitation and factors associated with providers' knowledge and skills: a cross-sectional study in Afghanistan. BMC Pediatrics. 2013;13(1):140-.

40. Knoble SJP, A.;Koirala, B.;Ghimire, L. Measuring the quality of rural-based, government health care workers in Nepal. Internet Journal of Allied Health Sciences & Practice. 2010;8(1):1-9 p.

41. Lobis SM, G.;Kamwendo, F.;McAuliffe, E.;Austin, J.;de Pinho, H. Expected to deliver: alignment of regulation, training, and actual performance of emergency obstetric care providers in Malawi and Tanzania. International journal of gynaecology and obstetrics: the official organ of the International Federation of Gynaecology and Obstetrics. 2011;115(3):322-7.

42. Makowiecka KA, E.;Izati, Y.;Ronsmans, C. Midwifery provision in two districts in Indonesia: how well are rural areas served? Health Policy & Planning. 2008;23(1):67-75 9p.

43. Malhotra S, Zodpey SP, Vidyasagaran AL, Sharma K, Raj SS, Neogi SB, et al. Assessment of Essential Newborn Care Services in Secondary-level Facilities from Two Districts of India. Journal of Health Population and Nutrition. 2014;32(1):130-41.

44. Mamba PP. Nurse-midwives knowledge & basis for decision-making on maternal reproductive high risk factors in pregnancy. Africa Journal of Nursing & Midwifery. 2000;2(2):58-62 5p.

45. Mangham-Jefferies L, Mathewos B, Russell J, Bekele A. How do health extension workers in Ethiopia allocate their time? Human resources for health. 2014;12(1):61- 1p.

46. Mansoor GF, Hashemy P, Gohar F, Wood ME, Ayoubi SF, Todd CS. Midwifery retention and coverage and impact on service utilisation in Afghanistan. Midwifery. 2013;29(10):1088-94 7p.

47. Mirkuzie AH, Sisay MM, Bedane MM. Standard basic emergency obstetric and neonatal care training in Addis Ababa; trainees reaction and knowledge acquisition. BMC Med Educ. 2014;14:201.

48. Nsemo ADJ, Mildred E.;Etifit, Rita E.;Mgbekem, Mary A.;Oyira, Emilia J. Clinical nurses' perception of continuing professional education as a tool for quality service delivery in public hospitals Calabar, Cross River State, Nigeria. Nurse Education in Practice. 2013;13(4):328-34 7p.

49. Nyango DDM, J. T.;Laabes, E. P.;Kigbu, J. H.;Buba, M. Skilled attendance: the key challenges to progress in achieving mdg-5 in north central nigeria...Millennium Development Goal. African Journal of Reproductive Health. 2010;14(2):129-38 10p.

50. Ogunlesi T, Dedeke O, Adekanmbi F, Fetuga B, Okeniyi A. Neonatal resuscitation: Knowledge and practice of nurses in western Nigeria. SAJCH South African Journal of Child Health. 2008;2(1):23-5.

51. Ojofeitimi EO, Asekun-Olarinmoye EO, Bamidele JO, Owolabi OO, Oladele EA. Poor knowledge of causes and prevention of stillbirths among health care providers. International Journal of Childbirth Education. 2009;24(4):26-9 4p.

52. Oladapo OT, Daniel OJ, Olatunji AO. Knowledge and use of the partograph among healthcare personnel at the peripheral maternity centres in Nigeria. J Obstet Gynaecol. 2006;26(6):538-41.

53. Oladapo OTF, A. O.;Loto, O. M.;Adegbola, O.;Akinola, O. I.;Alao, M. O.;Adeyemi, A. S. Active management of third stage of labour: a survey of providers' knowledge in southwest Nigeria. Arch Gynecol Obstet. 2009;280(6):945-52.

54. Opiah MMBO, Abosede;James Essien, Ekere;Monjok, Emmanuel. Knowledge and Utilization of the Partograph among Midwives in the Niger Delta Region of Nigeria. African Journal of Reproductive Health. 2012;16(1):125-32 8p.

55. Partamin K, Y. M.;Mungia, J.;Faqir, M.;Ansari, N.;Evans, C. Patterns in training, knowledge, and performance of skilled birth attendants providing emergency obstetric and newborn care in Afghanistan. INTERNATIONAL JOURNAL OF GYNECOLOGY & OBSTETRICS. 2012;119(2):125-9.

56. Parveen SQ, M. A.;Afroz, A.;Kolinsky, M.;Anwar, I. Improving the quality of nurse-midwives in Bangladesh: Addressing barriers of midwifery course in diploma in nursing and midwifery training. Dhaka, Bangladesh, International Centre for Diarrhoeal Disease Research, Bangladesh [ICDDR,B], Centre for Reproductive Health, 2011 Dec.; 2011.

57. Premadasa IGS, D.;Al-Jarallah, K. F.;Thalib, L. Frequency and confidence in performing clinical skills among medical interns in Kuwait. Medical Teacher. 2008;30(3):e60-5 1p.

58. Prytherch HK, D. C. V.;Leshabari, M. T.;Sauerborn, R.;Marx, M. Maternal and newborn healthcare providers in rural Tanzania: in-depth interviews exploring influences on motivation, performance and job satisfaction. Rural & Remote Health. 2012;12(3):1-15 p.

59. Sarfraz MH, Saima. Challenges in delivery of skilled maternal care - experiences of community midwives in Pakistan. BMC Pregnancy & Childbirth. 2014;14(1):59- 1p.

60. Saswata B, Omar F, Aubery RJ, Jaffer B, Michael W. Bridging the health gap in Uganda: the surgical role of the clinical officer. Afr Health Sci. 2005;5(1):86-9.

61. Schack SM, Elyas A, Brew G, Pettersson KO. Experiencing challenges when implementing Active Management of Third Stage of Labor (AMTSL): a qualitative study with midwives in Accra, Ghana. BMC Pregnancy & Childbirth. 2014;14(1):193- 1p.

62. Shaban IL, Nicky. A review of midwifery education curriculum documents in Jordan. Women & Birth. 2012;25(4):e47-55 1p.

63. Sharma BJ, Eva;Prakasamma, M.;Mavalankar, Dileep;Christensson, Kyllike. Midwifery scope of practice among staff nurses: A grounded theory study in Gujarat, India. Midwifery. 2013;29(6):628-36 9p.

64. Shimoda K, Leshabari S, Horiuchi S, Shimpuku Y, Tashiro J. Midwives' intrapartum monitoring process and management resulting in emergency referrals in Tanzania: a qualitative study. BMC Pregnancy & Childbirth. 2015;15(1):1-10 p.

65. Syamala TS. Do health worker female and traditional birth attendant equipped to provide primary health care in tribal areas? Evidences from Tribal Andhra Pradesh. Studies of Tribes and Tribes. 2004;2(2):119-24.

66. Traore M, Arsenault C, Schoemaker-Marcotte C, Coulibaly A, Huchon C, Dumont A, et al. Obstetric competence among primary healthcare workers in Mali. International journal of gynaecology and obstetrics: the official organ of the International Federation of Gynaecology and Obstetrics. 2014;126(1):50-5.

67. Ueno EA, Adetoro;Masenga, Gileard;Fimbo, Janeth;Msuya, Sia. Skilled Birth Attendants in Tanzania: A Descriptive Study of Cadres and Emergency Obstetric Care Signal Functions Performed. Maternal & Child Health Journal. 2015;19(1):155-69 15p.

68. Utz BS, Ghazna;Adegoke, Adetoro;Broek, Nynke. Definitions and roles of a skilled birth attendant: a mapping exercise from four South‐Asian countries. Acta Obstet Gynecol Scand. 2013;92(9):1063-9.

69. Yisma E, Dessalegn B, Astatkie A, Fesseha N. Knowledge and utilization of partograph among obstetric care givers in public health institutions of Addis Ababa, Ethiopia. BMC Pregnancy & Childbirth. 2013;13(1):17- 1p.

70. Zainullah PA, Nasratullah;Yari, Khalid;Azimi, Mahmood;Turkmani, Sabera;Azfar, Pashtoon;LeFevre, Amnesty;Mungia, Jaime;Gubin, Rehana;Young-Mi, Kim;Bartlett, Linda. Establishing midwifery in low-resource settings: Guidance from a mixed-methods evaluation of the Afghanistan midwifery education program. Midwifery. 2014;30(10):1056-62 7p.
